# Clinical use and toxicities of bortezomib in pediatric patients: a systematic review

**DOI:** 10.3389/fphar.2025.1661493

**Published:** 2025-08-06

**Authors:** Zachary C. LeBlanc, Averill Clapp, Samantha Kaplan, Carrie J. Shawber, June K. Wu

**Affiliations:** ^1^ Department of Surgery, Columbia University Irving Medical Center, New York, United States; ^2^ Department of Obstetrics & Gynecology, Columbia University Irving Medical Center, New York, United States

**Keywords:** bortezomib, adverse events, pediatric safety, systematic review, proteasome inhibitors

## Abstract

**Background:**

Proteasome inhibitors (PIs) are FDA-approved to treat adult malignancies. The PI, Bortezomib (BTZ), has been used off-label in pediatric patients but its safety profile in these patients has yet to be systematically assessed. We sought to review the pediatric safety profile of BTZ based on published clinical articles which we compared to publicly available adult safety data from the BTZ drug insert.

**Methods:**

PubMed and the Cochrane Database were searched up through September 2024. We included published clinical studies that reported adverse events (AEs) which included clinical trials, clinical studies (>10 patients), and clinical series/case reports (≤10 patients). Extracted pediatric safety data was compared to reported adult safety profile from the BTZ drug insert.

**Results:**

There was heterogeneity in reporting of different AEs and not all categories were comparable to published adult AEs.any studies were small case series or reports which did not allow for more quantitative analysis. Nevertheless, we found that pediatric patients treated with BTZ reported lower incidence of peripheral neuropathy and gastrointestinal toxicity compared to adults. Rates of bone marrow suppression and infection in pediatrics were comparable to or higher than those observed in adults These incidences were comparable or lower when pediatric patients with leukemia were excluded.

**Discussion:**

BTZ has an acceptable safety profile for use in pediatric patients. Antibacterial and antifungal prophylaxis should be considered given the high rate of infections.

## Introduction

Normal cellular homeostasis requires a balance of protein synthesis and degradation and is vital for cellular function and survivial (Teicher and Tomaszewski, 2015; Cromm and Crews, 2017). Proteostasis defects causing abnormal intracellular accumulation of misfolded or damaged proteins can cause cell death (Cvek and Dvorak, 2008; Teicher and Tomaszewski, 2015; Cao et al., 2021). One mechanism for protein degradation in the cell is the proteasome degradation (PD) pathway (Ciechanover, 2005; Cvek and Dvorak, 2008; Teicher and Tomaszewski, 2015; Cao et al., 2021), which is mediated by the 26S proteasome, consisting of a 20S core flanked by two 19S regulatory subunits (Teicher and Tomaszewski, 2015; Cromm and Crews, 2017).

Studies have shown that cancer cells are more reliant on the PD pathway to maintain homeostasis and thus more sensitive to the effects of proteasome inhibition (Delic et al., 1998; Adams, 2004; Nawrocki et al., 2005; Crawford et al., 2009; Crawford et al., 2011). Proteasome inhibitors (PIs) comprise a class of drugs that specifically target the 20S subunit of the proteasome (Teicher and Tomaszewski, 2015; Wang et al., 2021) and preferentially target cancer cells with proteostasis defects, leading to exacerbated cytoplasmic protein accumulation and cell death (Delic et al., 1998; Nawrocki et al., 2005; Teicher and Tomaszewski, 2015). Five PIs are currently in clinical use: bortezomib (BTZ), carfilzomib, marizomib, oprozomib, and ixazomib (Richardson et al., 2003b; Chauhan et al., 2005; Piva et al., 2008; Chauhan et al., 2010; Potts et al., 2011; Muz et al., 2016; Perel et al., 2016). One PI, delanzomib, is currently in clinical trials (Vogl et al., 2017).

BTZ is a first-generation PI that binds the 20S proteasome and approved in 2003 to treat refractory multiple myeloma (MM) in adults and is currently FDA approved for adult use in *de novo* and relapsed/refractory MM and relapsed/refractory mantle cell lymphoma (Raedler, 2015). While not FDA-approved for pediatric patients, BTZ has been prescribed off-label in the pediatric setting for both malignant and non-malignant indications. This systematic review serves to systematically review the safety profile of BTZ in pediatric patients from the published literature and compared to adults. The goal is to identify strategies for the safe and effective use of PIs in pediatric patients.

## Patients and methods

### Search strategy

A search of PubMed and the Cochrane Database of Systematic Reviews was performed in September of 2024. Search terms were separated by Boolean operators as follows: (“bortezomib”) AND (“pediatric” OR “children”). Article types were “adaptive clinical trials”, “case reports”, “clinical studies”, “clinical trials phase 1-4”, “controlled clinical trials”, “randomized controlled trials”, and “multi-center studies”. References of selected articles were searched for additional studies. In July 2025, clinical trial registries were searched on clinicaltrials.gov using the terms “bortezomib” and “pediatric”; “bortezomib” and “children”. Those registries with results posted were reviewed for inclusion or exclusion into our analysis. Findings were compared to the drug insert provided by Takeda for adult adverse events (AEs) where available (Takeda Pharmaceuticals, 2022).

### Inclusion/exclusion criteria

Studies included for analysis are those that reported on pediatric patients ≤18 years of age treated with BTZ for all indication, as a single agent or in combination with other therapeutics. Patients ≤8 years of age were further stratified and defined as young pediatric patients for analysis. Studies were excluded if they met any of the following criteria: (1) review articles, (2) preclinical studies, (3) clinical studies that included adult patients where pediatric data could not be separated, (4) trial design only, (5) patient reported outcomes, (6) did not use BTZ, and (7) not available in English.

### Article selection and data extraction

Full-text articles from the initial search were reviewed independently by two authors (Z.C.L., A.C.). Data extraction was performed independently by each author and compared for accuracy prior to inclusion. Data collected included study type, date of publication, number of enrolled patients, number of patients assessed for AEs, treatment indication, co-treatment, grade and incidence of complications, dose-limiting toxicities, and deaths attributable to BTZ.

### Toxicity classification

The CTCAE system was used for toxicity classification. Identified AEs were classified by organ systems and included the neurological, bone marrow, infectious, respiratory, cardiac, and gastrointestinal systems. Toxicities are reported here as individual CTCAE terms or grouped by broader organ system category based on reporting of source documents. Toxicities from the same CTCAE group were tabulated as single event when it was clear that the toxicities occurred in a single patient. Dose limiting toxicity (DLT) was defined as AEs that required dose reduction, temporary or permanent cessation of BTZ. Treatment-related mortality was defined as death within 30 days of last BTZ dose that was possibly attributed to BTZ.

### Synthesis

Data was collected and analyzed using Microsoft Excel (Version 16.0). Studies that did not report on certain categories of AEs were excluded from the corresponding tabulations of patients who experienced these complications. Finally, pediatric AEs incidences were compared to those reported for adults in the commercial packaging insert for BTZ.

## Results

### Study selection

**FIGURE 1:**
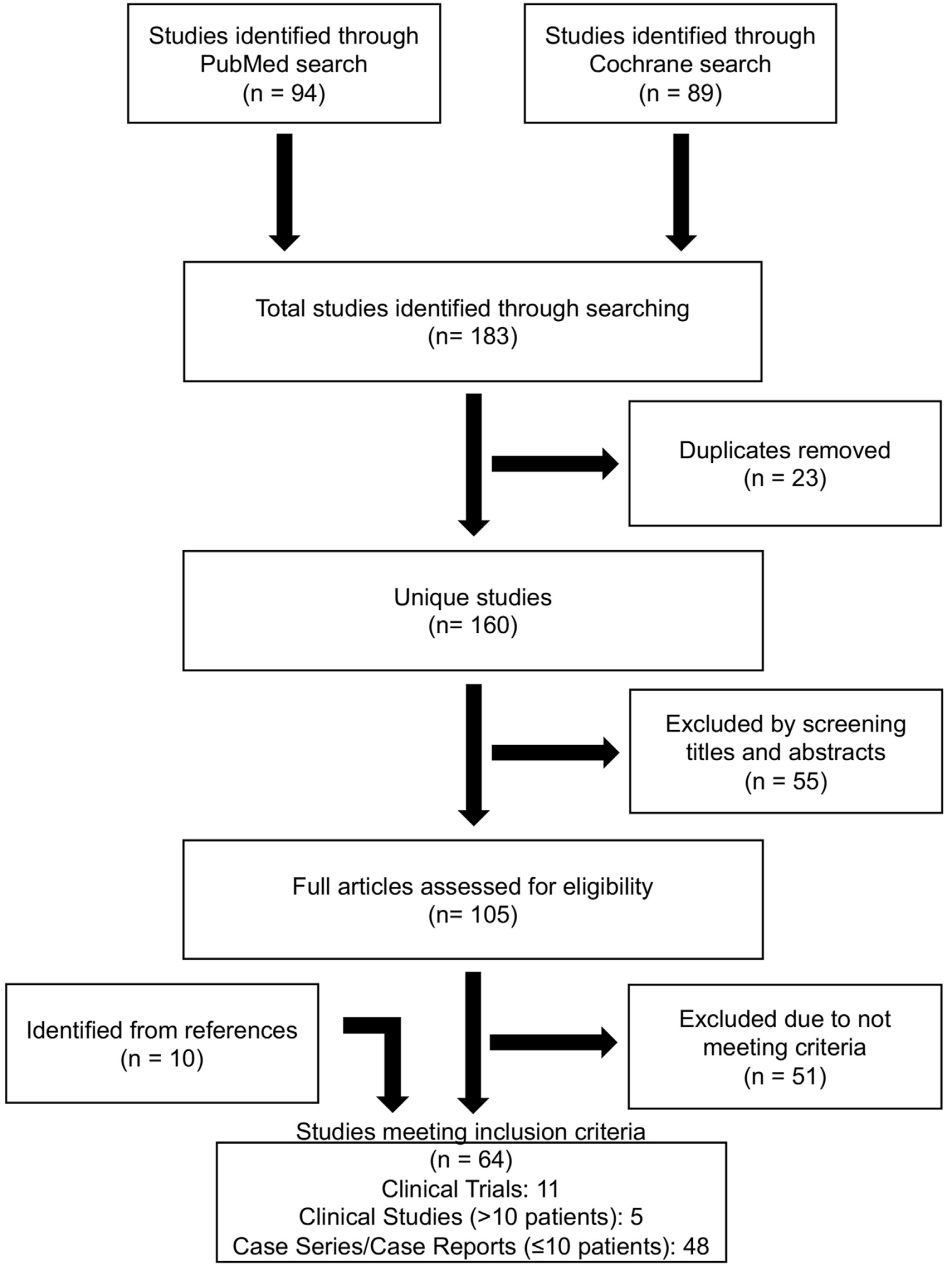
Database search strategy. PRISMA flowchart of database search, inclusion and exclusion criteria to result in eligible publications used for systematic review.

Search criteria across PubMed and Cochrane databases identified 183 articles of which 23 were duplicates. Screening the abstract resulted in the exclusion of 22 that were trial registrations, 31 conference proceedings, and 2 articles not available in English (Figure 1). 105 full text articles were assessed for eligibility. An additional 25 were excluded because they combined both adult and pediatric patient data from which pediatric data could not be extracted, 8 that did not use BTZ, 8 containing only laboratory data, 9 with no discussion of adverse events, and 1 that addressed trial design theories with no reported patient interventions (n = 54). An additional 10 studies were found within references of identified articles. Articles were downloaded and reviewed in their entirety for data extraction and analysis. In total, 64 articles met inclusion criteria (Figure 1).

Search criteria across registered clinical trials matching “bortezomib” and “pediatric” returned 26 trials and “bortezomib” and “children” returned 10 trials. After removing duplicates 10 unique trials had posted results. Of trials with results, 3 trials were published and already included in our analysis (Figure 1), 6 trials encompassed both pediatric and adult patients and pediatric data could not be extracted, and 1 trial included only adult (≥18 years old) patients. Thus, clinical trials registry did not yield additional analyzable data for this review.

### Study types and treatment indications

The studies that met inclusion criteria included 11 clinical trials, 5 clinical studies (>10 patients), and 48 case series/case reports (≤10 patients) (Supplementary Tables S1–S3). Clinical trials included phase 1 (n = 9), phase 2 (n = 1), and phase 3 (n = 1) trials. Treatment indications included solid tumors, new/relapsed acute lymphoblastic leukemia (ALL) and acute myeloid leukemia (AML) (Supplementary Table S1).

Treatment indications for the clinical studies and case series/case reports included B-ALL, T-ALL, solid tumors, MM, antibody-mediated transplantation rejection, proliferative glomerulonephritis, autoimmune cytopenias after hematopoietic stem cell or intestinal transplant, refractory Evan’s syndrome, refractory TTP, refractory anti-NMDA encephalitis, complications secondary to ERT for Pompe’s disease, and heparin induced thrombocytopenia (Supplementary Tables S2, S3). BTZ was used as a single agent in 2 clinical trials and 2 case series but was more frequently administered with other drugs including chemotherapeutic agents and corticosteroids (Supplementary Tables S1–S3).

### Peripheral neuropathy and other neurological AEs

**FIGURE 2:**
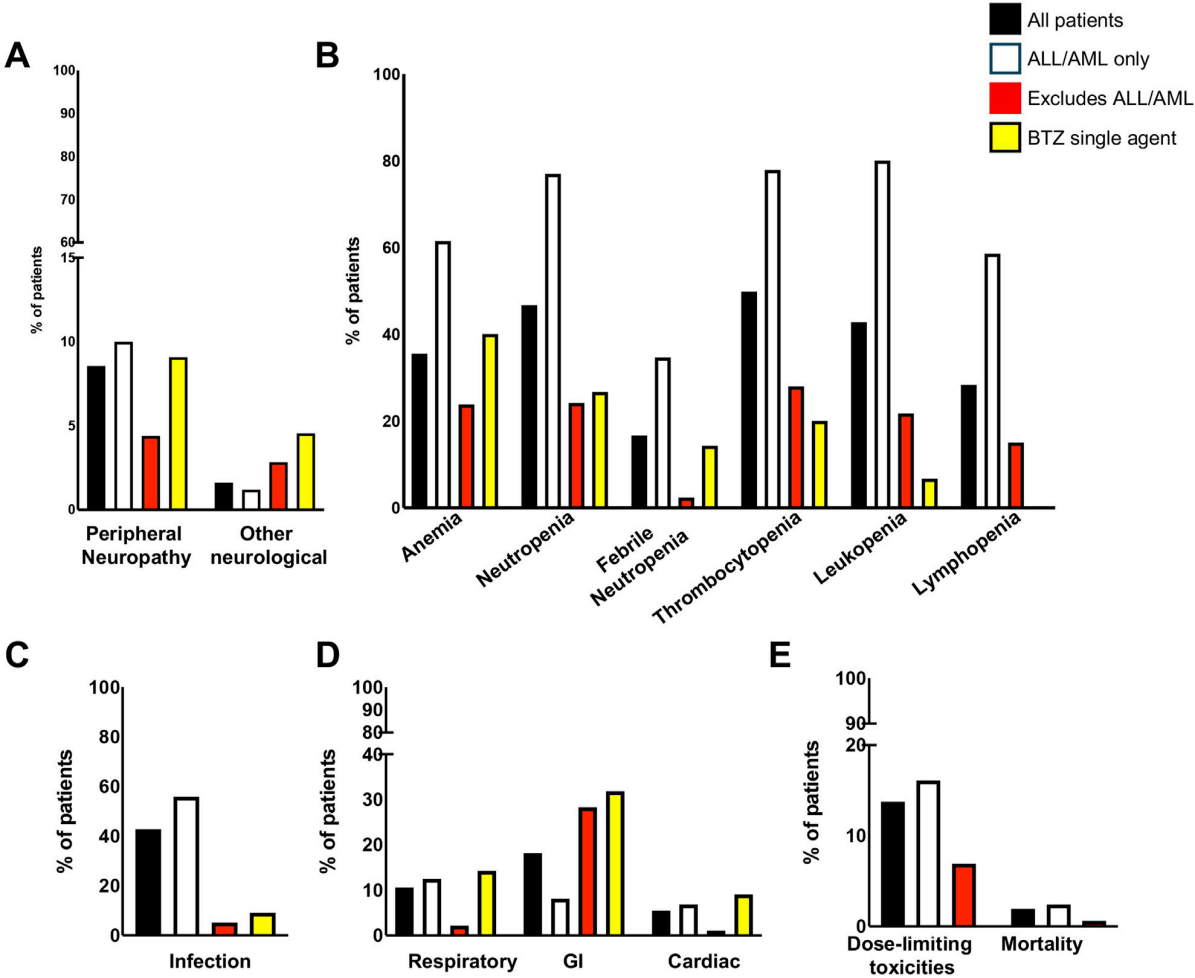
Incidence of adverse events by patient groups. **(A)** Neurological symptoms; **(B)** Bone marrow indices; **(C)** Infection; **(D)** Respiratory, GI, and Cardiac; **(E)** Dose limiting toxicities and mortality. GI, gastrointestinal.

Dose-dependent, reversible peripheral neuropathy (PN) is a known BTZ AE (Richardson et al., 2005; Argyriou et al., 2008; San Miguel et al., 2008; Moreau et al., 2011; Robak et al., 2018). In the BTZ drug insert, 38% of adult patients receiving BTZ experienced PN, with 11% experiencing ≥ grade 3 toxicity, leading to 8% of all patients to discontinue treatment (Takeda Pharmaceuticals, 2022). The overall incidence of PN in pediatric patients treated with BTZ was 8.56%, with 3.17% experiencing ≥ grade 3 toxicity (Tables 1, 2; Figure 2A). In young pediatric patients, defined as patients 8 years or younger, the overall incidence was 2.46% (Table 3). ALL and AML patients had a 10% incidence of PN. When ALL and AML were excluded, only 4.40% of pediatric patients and no young pediatric patients experienced PN (Tables 1, 3). Overall, pediatric patients experienced a lower incidence of PNs than adults, and PN improved or resolved completely after BTZ cessation.

**TABLE 1:** Summary of toxicities of all grades in pediatric patients treated with bortezomib.

Indication	Evaluable patients	# of adverse events	% of patients experiencing complication, all grades
Peripheral Neuropathy			
ALL	169	24	14.20%
AML	291	22	7.56%
Solid tumors	54	6	11.11%
Transplant	68	1	1.47%
Autoimmune cytopenias	20	0	0.00%
Anti-NMDA encephalitis	4	0	0.00%
Other	13	0	0.00%
Total	**619**	53	**8.56%**
ALL/AML only	**460**	46	**10.00%**
Total excluding ALL/AML	**159**	7	**4.40%**
Other Neurological			
ALL	128	5	3.91%
AML	287	0	0.00%
Solid tumors	36	1	2.78%
Transplant	68	3	4.41%
Autoimmune cytopenias	20	0	0.00%
Anti-NMDA encephalitis	4	0	0.00%
Other	13	0	0.00%
Total	**556**	9	**1.62%**
ALL/AML only	**415**	5	**1.20%**
Total excluding ALL/AML	**141**	4	**2.84%**
Anemia			
ALL	70	43	61.43%
AML	0	0	
Solid tumors	52	31	59.62%
Transplant	66	6	9.09%
Autoimmune cytopenias	20	0	0.00%
Anti-NMDA encephalitis	4	0	0.00%
Other	13	0	0.00%
Total	**225**	80	**35.56%**
ALL only	**70**	43	**61.43%**
Total excluding ALL	**155**	37	**23.87%**
Neutropenia			
ALL	117	90	76.92%
AML	0	0	
Solid tumors	52	33	63.46%
Transplant	68	4	5.88%
Autoimmune cytopenias	20	0	0.00%
Anti-NMDA encephalitis	4	1	25.00%
Other	13	0	0.00%
Total	**274**	128	**46.72%**
ALL only	**117**	90	**76.92%**
Total excluding ALL	**157**	38	**24.20%**
Febrile Neutropenia			
ALL	97	31	31.96%
AML	4	4	100.00%
Solid tumors	22	1	4.55%
Transplant	68	1	1.47%
Autoimmune cytopenias	20	1	5.00%
Anti-NMDA encephalitis	4	0	0.00%
Other	13	0	0.00%
Total	**228**	38	**16.67%**
ALL/AML only	**101**	35	**34.65%**
Total excluding ALL/AML	**127**	3	**2.36%**
Thrombocytopenia			
ALL	122	95	77.87%
AML	0	0	
Solid tumors	52	32	61.54%
Transplant	68	9	13.24%
Autoimmune cytopenias	20	3	15.00%
Anti-NMDA encephalitis	4	0	0.00%
Other	13	0	0.00%
Total	**279**	139	**49.82%**
ALL only	**122**	95	**77.87%**
Total excluding ALL	**157**	44	**28.03%**
Leukopenia			
ALL	70	56	80.00%
AML	0	0	
Solid tumors	52	26	50.00%
Transplant	35	0	0.00%
Autoimmune cytopenias	20	1	5.00%
Anti-NMDA encephalitis	4	0	0.00%
Other	13	0	0.00%
Total	**194**	83	**42.78%**
ALL only	**70**	56	**80.00%**
Total excluding ALL	**124**	27	**21.77%**
Lymphopenia			
ALL	41	24	58.54%
AML	0	0	
Solid tumors	21	11	52.38%
Transplant	35	3	8.57%
Autoimmune cytopenias	20	0	0.00%
Anti-NMDA encephalitis	4	0	0.00%
Other	13	0	0.00%
Total	**134**	38	**28.36%**
ALL only	**41**	24	**58.54%**
Total excluding ALL	**93**	14	**15.05%**
Infection			
ALL	169	54	31.95%
AML	291	203	69.76%
Solid tumors	54	0	0.00%
Transplant	68	6	8.82%
Autoimmune cytopenias	20	1	5.00%
Anti-NMDA encephalitis	4	0	0.00%
Other	13	1	7.69%
Total	**619**	265	**42.81%**
ALL/AML only	**460**	257	**55.87%**
Total excluding ALL/AML	**159**	8	**5.03%**
Respiratory toxicities, excluding those attributable to infection			
ALL	121	4	3.31%
AML	287	47	16.38%
Solid tumors	21	1	4.76%
Transplant	33	1	3.03%
Autoimmune cytopenias	20	0	0.00%
Anti-NMDA encephalitis	4	0	0.00%
Other	13	0	0.00%
Total	**499**	53	**10.62%**
ALL/AML only	**408**	51	**12.50%**
Total excluding ALL/AML	**91**	2	**2.20%**
GI			
ALL	156	13	8.33%
AML	4	0	0.00%
Solid tumors	54	33	61.11%
Transplant	68	9	13.24%
Autoimmune cytopenias	20	3	15.00%
Anti-NMDA encephalitis	4	0	0.00%
Other	13	0	0.00%
Total	**319**	58	**18.18%**
ALL/AML only	**160**	13	**8.13%**
Total excluding ALL/AML	**159**	45	**28.30%**
Cardiac			
ALL	21	2	9.52%
AML	287	17	5.92%
Solid tumors	19	1	5.26%
Transplant	35	0	0.00%
Autoimmune cytopenias	20	0	0.00%
Anti-NMDA encephalitis	4	0	0.00%
Other	13	0	0.00%
Total	**399**	22	**5.51%**
ALL/AML only	**308**	21	**6.82%**
Total excluding ALL/AML	**91**	1	**1.10%**
Dose-limiting toxicities			
ALL	169	9	5.33%
AML	291	65	22.34%
Solid tumors	54	7	12.96%
Transplant	68	2	2.94%
Autoimmune cytopenias	20	2	10.00%
Anti-NMDA encephalitis	4	0	0.00%
Other	13	0	0.00%
Total	**619**	85	**13.73%**
ALL/AML only	**460**	74	**16.09%**
Total excluding ALL/AML	**159**	11	**6.92%**
Mortality			
ALL	169	11	6.51%
AML	291	0	0.00%
Solid tumors	54	0	0.00%
Transplant	68	1	1.47%
Autoimmune cytopenias	27	0	0.00%
Anti-NMDA encephalitis	4	0	0.00%
Other	13	0	0.00%
Total	**626**	12	**1.92%**
ALL/AML only	**460**	11	**2.39%**
Total excluding ALL/AML	**166**	1	**0.60%**

^1^Incidents of reported infections, not individual patients.

Acute lymphoblastic leukemia (ALL); acute myeloid leukemia (AML); not reported (NR); anti-NMDA, Anti-N-methyl-d-aspartate.

**TABLE 2:** Summary of grade 3+ toxicities in pediatric patients treated with bortezomib.

Indication	Evaluable patients	# of adverse events	% of patients experiencing complication, grade 3+
Peripheral Neuropathy			
ALL	159	8	5.03%
AML	0		
Solid tumors	52	2	3.85%
Transplant	67	0	0.00%
Autoimmune cytopenias	20	0	0.00%
Anti-NMDA encephalitis	4	0	0.00%
Other	13	0	0.00%
Total	315	10	**3.17%**
Other Neurological			
ALL	128	4	3.13%
AML	0		
Solid tumors	36	0	0.00%
Transplant	35	0	0.00%
Autoimmune cytopenias	20	0	0.00%
Anti-NMDA encephalitis	4	0	0.00%
Other	13	0	0.00%
Total	236	4	**1.69%**
Anemia			
ALL	70	38	54.29%
AML	0		
Solid tumors	51	7	13.73%
Transplant	32	2	6.25%
Autoimmune cytopenias	20	0	0.00%
Anti-NMDA encephalitis	4	0	0.00%
Other	13	0	0.00%
Total	190	47	**24.74%**
Neutropenia			
ALL	116	89	76.72%
AML	0		
Solid tumors	51	14	27.45%
Transplant	35	1	2.86%
Autoimmune cytopenias	20	0	0.00%
Anti-NMDA encephalitis	3	0	0.00%
Other	13	0	0.00%
Total	238	104	**43.70%**
Febrile Neutropenia			
ALL	70	17	24.29%
AML	0		
Solid tumors	22	1	4.55%
Transplant	35	0	0.00%
Autoimmune cytopenias	13	0	0.00%
Anti-NMDA encephalitis	4	0	0.00%
Other	13	0	0.00%
Total	157	18	**11.46%**
Thrombocytopenia			
ALL	117	92	78.63%
AML	0		
Solid tumors	51	14	27.45%
Transplant	30	1	3.33%
Autoimmune cytopenias	19	1	5.26%
Anti-NMDA encephalitis	4	0	0.00%
Other	13	0	0.00%
Total	234	108	**46.15%**
Leukopenia			
ALL	70	56	80.00%
AML	0		
Solid tumors	51	7	13.73%
Transplant	35	0	0.00%
Autoimmune cytopenias	18	0	0.00%
Anti-NMDA encephalitis	4	0	0.00%
Other	13	0	0.00%
Total	191	63	**32.98%**
Lymphopenia			
ALL	41	24	58.54%
AML	0		
Solid tumors	20	5	25.00%
Transplant	34	2	5.88%
Autoimmune cytopenias	20	0	0.00%
Anti-NMDA encephalitis	4	0	0.00%
Other	13	0	0.00%
Total	132	31	**23.48%**
Infection			
ALL	147	48	32.65%
AML	0		
Solid tumors	54	0	0.00%
Transplant	54	0	0.00%
Autoimmune cytopenias	13	0	0.00%
Anti-NMDA encephalitis	4	0	0.00%
Other	11	0	0.00%
Total	283	48	**16.96%**
Respiratory toxicities, excluding those attributable to infection			
ALL	117	3	2.56%
AML	0		
Solid tumors	21	0	0.00%
Transplant	31	0	0.00%
Autoimmune cytopenias	20	0	0.00%
Anti-NMDA encephalitis	4	0	0.00%
Other	13	0	0.00%
Total	206	3	**1.46%**
GI			
ALL	112	8	7.14%
AML	0		
Solid tumors	51	5	9.80%
Transplant	28	0	0.00%
Autoimmune cytopenias	12	0	0.00%
Anti-NMDA encephalitis	4	0	0.00%
Other	13	0	0.00%
Total	220	13	**5.91%**
Cardiac			
ALL	21	2	9.52%
AML	0		
Solid tumors	4	0	0.00%
Transplant	35	0	0.00%
Autoimmune cytopenias	20	0	0.00%
Anti-NMDA encephalitis	4	0	0.00%
Other	13	0	0.00%
Total	97	2	**2.06%**

^1^Incidents of reported infections, not individual patients.

Acute lymphoblastic leukemia (ALL); acute myeloid leukemia (AML); not reported (NR); anti-NMDA, Anti-N-methyl-d-aspartate.

**TABLE 3:** Summary of toxicities in pediatric patients age ≤8 treated with bortezomib.

Indication	Evaluable patients	# of adverse events	% of patients experiencing complication, all grades
Peripheral Neuropathy			
ALL	11	3	27.27%
AML	82	0	0.00%
Solid tumors	0		
Transplant	14	0	0.00%
Autoimmune cytopenias	5	0	0.00%
Anti-NMDA encephalitis	2	0	0.00%
Other	8	0	0.00%
Total	**122**	3	**2.46%**
ALL/AML only	**93**	3	**3.23%**
Total excluding ALL/AML	**29**	0	**0.00%**
Other Neurological			
ALL	3	0	0.00%
AML	81	1	1.23%
Solid tumors	0		
Transplant	14	0	0.00%
Autoimmune cytopenias	5	0	0.00%
Anti-NMDA encephalitis	2	0	0.00%
Other	8	0	0.00%
Total	**113**	1	**0.88%**
ALL/AML only	**84**	1	**1.19%**
Total excluding ALL/AML	**29**	0	**0.00%**
Anemia			
ALL	3	0	0.00%
AML	0		
Solid tumors	0		
Transplant	14	1	7.14%
Autoimmune cytopenias	5	0	0.00%
Anti-NMDA encephalitis	2	0	0.00%
Other	8	0	0.00%
Total	**32**	1	**3.13%**
ALL/AML only	**3**	0	**0.00%**
Total excluding ALL	**29**	1	**3.45%**
Neutropenia			
ALL	3	0	0.00%
AML	0		
Solid tumors	0		
Transplant	14	0	0.00%
Autoimmune cytopenias	5	0	0.00%
Anti-NMDA encephalitis	2	1	50.00%
Other	8	0	0.00%
Total	**32**	1	**3.13%**
ALL/AML only	**3**	0	**0.00%**
Total excluding ALL	**29**	1	**3.45%**
Febrile Neutropenia			
ALL	6	3	50.00%
AML	1	1	100.00%
Solid tumors	0		
Transplant	14	0	0.00%
Autoimmune cytopenias	5	0	0.00%
Anti-NMDA encephalitis	2	0	0.00%
Other	8	0	0.00%
Total	**36**	4	**11.11%**
ALL/AML only	**7**	4	**57.14%**
Total excluding ALL	**29**	0	**0.00%**
Thrombocytopenia			
ALL	3	0	0.00%
AML	0		
Solid tumors	0		
Transplant	14	1	7.14%
Autoimmune cytopenias	5	1	20.00%
Anti-NMDA encephalitis	2	0	0.00%
Other	8	0	0.00%
Total	**32**	2	**6.25%**
ALL/AML only	**3**	0	**0.00%**
Total excluding ALL	**29**	2	**6.90%**
Leukopenia			
ALL	3	0	0.00%
AML	0		
Solid tumors	0		
Transplant	14	0	0.00%
Autoimmune cytopenias	5	1	20.00%
Anti-NMDA encephalitis	2	0	0.00%
Other	8	0	0.00%
Total	**32**	1	**3.13%**
ALL/AML only	**3**	0	**0.00%**
Total excluding ALL	**29**	1	**3.45%**
Lymphopenia			
ALL	3	0	0.00%
AML	0		
Solid tumors	0		
Transplant	14	1	7.14%
Autoimmune cytopenias	5	0	0.00%
Anti-NMDA encephalitis	2	0	0.00%
Other	8	0	0.00%
Total	**32**	1	**3.13%**
ALL/AML only	**3**	0	**0.00%**
Total excluding ALL	**29**	1	**3.45%**
Infection^a^			
ALL	11	2	18.18%
AML	82	48	58.54%
Solid tumors	0		
Transplant	14	3	21.43%
Autoimmune cytopenias	5	0	0.00%
Anti-NMDA encephalitis	2	0	0.00%
Other	8	1	12.50%
Total	**122**	54	**44.26%**
ALL/AML only	**93**	50	**53.76%**
Total excluding ALL/AML	**29**	4	**13.79%**
Respiratory toxicities, excluding those attributable to infection			
ALL	3	0	0.00%
AML	81	13	16.05%
Solid tumors	0		
Transplant	14	1	7.14%
Autoimmune cytopenias	5	0	0.00%
Anti-NMDA encephalitis	2	0	0.00%
Other	8	0	0.00%
Total	**113**	14	**12.39%**
ALL/AML only	**84**	13	**15.48%**
Total excluding ALL/AML	**29**	1	**3.45%**
GI			
ALL	11	2	18.18%
AML	1	0	0.00%
Solid tumors	0		
Transplant	14	2	14.29%
Autoimmune cytopenias	5	1	20.00%
Anti-NMDA encephalitis	2	0	0.00%
Other	8	0	0.00%
Total	**41**	5	**12.20%**
ALL/AML only	**12**	2	**16.67%**
Total excluding ALL/AML	**29**	3	**10.34%**
Cardiac			
ALL	0	0	9.52%
AML	81	1	1.23%
Solid tumors	0		
Transplant	14	0	0.00%
Autoimmune cytopenias	5	0	0.00%
Anti-NMDA encephalitis	2	0	0.00%
Other	8	0	0.00%
Total	**110**	1	**0.91%**
ALL/AML only	**81**	1	**1.23%**
Total excluding ALL/AML	**29**	0	**0.00%**
Dose-limiting toxicities			
ALL	11	0	0.00%
AML	82	12	14.63%
Solid tumors	0		
Transplant	14	0	0.00%
Autoimmune cytopenias	5	1	20.00%
Anti-NMDA encephalitis	2	0	0.00%
Other	8	0	0.00%
Total	**122**	13	**10.66%**
ALL/AML only	**93**	12	**12.90%**
Total excluding ALL/AML	**29**	1	**3.45%**
Mortality			
ALL	11	0	0.00%
AML	82	0	0.00%
Solid tumors	0		
Transplant	14	1	7.14%
Autoimmune cytopenias	5	0	0.00%
Anti-NMDA encephalitis	2	0	0.00%
Other	8	0	0.00%
Total	**122**	1	**0.82%**
ALL/AML only	**93**	0	**0.00%**
Total excluding ALL/AML	**29**	1	**3.45%**

^a^ Incidents of reported infections, not individual patients.

Acute lymphoblastic leukemia (ALL); acute myeloid leukemia (AML); not reported (NR); anti-NMDA, Anti-N-methyl-d-aspartate.

Other neurological AEs have also been reported in adults. Headaches were reported in 15% of patients, with <1% experiencing ≥ grade 3 toxicity. Transient ischemic attack, hemorrhagic stroke, coma, and Posterior Reversible Encephalopathy Syndrome were also reported although the incidences were not available (Takeda Pharmaceuticals, 2022). The overall incidence of other neurological AEs in pediatric patients treated with BTZ was 1.62%, with 1.69% of evaluable patients experiencing ≥ grade 3 toxicity (Tables 1, 2). Toxicities included headache, confusion, depressed level of consciousness, and seizures. In young pediatric patients, the overall incidence was 0.88% (Table 3). ALL and AML patients had a 1.2% incidence of other neurological AEs. When ALL and AML patients were excluded, 2.84% of pediatric patients and no young pediatric patients experienced other neurological AEs (Tables 1, 3; Figure 2A). Pediatric patients experienced fewer neurological AEs than adults.

### Bone marrow toxicity

Bone marrow suppression has been reported for PIs in adults (Richardson et al., 2003a; Jagannath et al., 2004; Murai et al., 2014; Kim et al., 2015). Reported bone marrow toxicities in the BTZ clinical trial data for Mantle Cell Lymphoma and Multiple Myeloma included anemia, neutropenia/febrile neutropenia, thrombocytopenia, leukopenia, and lymphopenia (Takeda Pharmaceuticals, 2022). Pediatric studies reviewed in this systematic review similarly reported high incidences of bone marrow suppression.

The incidence of anemia in adults was 18%, with 6% experiencing ≥ grade 3 toxicity (Takeda Pharmaceuticals, 2022). Pediatric incidence was higher than adults at 35.56%, with 24.74% experiencing ≥ grade 3 toxicity (Tables 1, 2; Figure 2B). In young pediatric patients, the incidence of any grade anemia was 3.13% (Table 3). The majority of ALL patients (61.43%) experienced anemia. When ALL patients were excluded, 23.87% of pediatric patients and 3.45% of young pediatric patients experienced anemia (Tables 1, 3; Figure 2B).

The rate of neutropenia in adults was 15%, with 10% experiencing ≥ grade 3 toxicity (Takeda Pharmaceuticals, 2022). Pediatric incidence of neutropenia was higher at 46.62%, with 43.70% experiencing ≥ grade 3 toxicity (Tables 1, 2; Figure 2B). In young pediatric patients, the incidence of any grade neutropenia was 3.13% (Table 3). The majority of ALL patients (76.92%) experienced neutropenia. When patients with ALL were excluded, 24.20% of pediatric patients and 3.45% of young pediatric patients experienced neutropenia (Tables 1, 3; Figure 2B).

Febrile neutropenia, not described in the adult drug insert, was observed in 16.67% of pediatric patients, with 11.46% experiencing ≥ grade 3 toxicity (Tables 1, 2; Figure 2B). In young pediatric patients, the incidence of any grade febrile neutropenia was 11.11% (Table 3). ALL and AML patients had a 34.65% incidence of febrile neutropenia. When patients with ALL and AML were excluded, 2.36% of all pediatric patients and no young pediatric patients experienced febrile neutropenia (Tables 1, 3; Figure 2B).

The incidence of thrombocytopenia in adults was 32%, with 25% experiencing ≥ grade 3 toxicity (Takeda Pharmaceuticals, 2022). Pediatric incidence was higher at 49.82%, with 46.15% experiencing ≥ grade 3 toxicity (Tables 1, 2; Figure 2B). In young pediatric patients, the incidence of any grade thrombocytopenia was 6.25% (Table 3). The majority of ALL patients (77.87%) experienced thrombocytopenia. When patients with ALL were excluded, 28.03% of pediatric patients and 6.90% of young pediatric patients experienced thrombocytopenia (Tables 1, 3; Figure 2B).

While not reported in the combined adult drug insert (Takeda Pharmaceuticals, 2022), Robak et al. reported a rate of leukopenia in adults with previously untreated mantle cell lymphoma of 48% for all grades, with 43% experiencing ≥ grade 3 toxicity (Robak et al., 2018). Pediatric incidence was comparable at 42.78%, with 32.98% experiencing ≥ grade 3 toxicity (Tables 1, 2; Figure 2B). In young pediatric patients, the incidence of any grade leukopenia was 3.13% (Table 3). The majority of ALL patients (80%) experienced leukopenia. When patients with ALL were excluded, 21.77% of pediatric patients and 3.45% of young pediatric patients experienced leukopenia (Tables 1, 3; Figure 2B).

Similarly, Robak et al. found that 28% adults with previously untreated mantle cell lymphoma experienced lymphopenia, with 25% experiencing ≥ grade 3 toxicity (Robak et al., 2018). Pediatric lymphopenia incidence was comparable, with an overall incidence of 28.36%, with 23.48% experiencing ≥ grade 3 toxicity (Tables 1, 2; Figure 2B). In young pediatric patients, the incidence lymphopenia was 3.13% (Table 3). The majority of ALL patients (58.54%) experienced lymphopenia. When ALL patients were excluded, 15.05% of pediatric patients and 3.45% young pediatric patients experienced lymphopenia (Tables 1, 3).

### Infections

Infection is a known BTZ AE in both adult and pediatric populations. Infectious complications including bacterial, viral, and fungal infections in BTZ-treated adults. Of these, only the incidence of pneumonia (8%) and herpes zoster (11%) were reported (Takeda Pharmaceuticals, 2022). Infection of any kind occurred in 42.81% of pediatric patients during BTZ treatment (Table 1; Figure 2C), with 16.96% experiencing ≥ grade 3 toxicity (Table 2). Reported infection types ranged from mild sinusitis to severe life-threatening pulmonary aspergillosis, bacteremia or sepsis (Supplementary Tables S4–S6). In young pediatric patients, the incidence of any grade infection was 44.26% (Table 3). The majority of ALL and AML patients (55.87%) experienced lymphopenia. When these patients with ALL and AML were excluded, 5.03% of pediatric patients and 13.79% of young pediatric patients experienced infection (Tables 1, 3; Figure 2C).

### Respiratory toxicity

Respiratory toxicity is a rare but potentially serious complication of BTZ in adults, ranging in severity from asthma-like symptoms or dyspnea to rapidly progressive pulmonary fibrosis (Saglam et al., 2020), though the incidence of milder respiratory toxicity in BTZ-treated adults treated is unknown. Respiratory events reported in pediatric studies included hypoxia, respiratory failure, and ARDS, which were pooled into a single category because of differences in reporting across studies. The overall incidence of respiratory toxicity was 10.62%, with 1.46% experiencing ≥ grade 3 toxicity (Tables 1, 2; Figure 2D). In young pediatric patients, the incidence was 12.39% (Table 3). ALL and AML patients had a 12.50% incidence of respiratory AEs. When patients with AML and ALL were excluded, 2.20% of pediatric patients and 3.45% of young pediatric patients experienced respiratory toxicity (Tables 1, 3; Figure 2D).

### Gastrointestinal toxicity

Gastrointestinal (GI) toxicities are known AEs of BTZ treatment owing to its effect on rapidly dividing GI epithelial cells. In BTZ-treated adults, 20% had anorexia, 28% had vomiting, and 46% experienced diarrhea; 7%, 2%, and 4% experienced these ≥ grade 3 toxicities, respectively (Takeda Pharmaceuticals, 2022). GI toxicities for pediatrics were categorized differently than in adults (Takeda Pharmaceuticals, 2022) and thus direct comparison was not possible. Nevertheless, pediatric incidence of GI AE appeared lower. Reported GI toxicities in pediatric patients included abdominal pain, typhlitis/colitis, nausea, vomiting, anorexia, diarrhea, mucositis, enterocolitis, ileus, and pancreatitis. When multiple GI toxicities occurred in a single patient, they are reported as a single GI AE. The overall incidence of GI AEs was 18.18%, with 5.91% experiencing ≥ grade 3 toxcity (Tables 1, 2; Figure 2D). In young pediatric patients, the rate of any grade GI toxicity was 12.20% (Table 3). ALL and AML patients had a 8.13% incidence of GI toxicities. When patients with ALL and AML were excluded, 28.30% of pediatric patients and 10.34% of young pediatric patients experienced GI toxicity (Tables 1, 3; Figure 2D).

### Cardiac toxicities

Cardiac toxicity has been described in the drug insert as a potential BTZ AE. The adult incidence of treatment-related cardiac conditions is at 8% and includes new or worsening heart failure, decreased left ventricular ejection fraction, ischemic symptoms/myocardial infarction, arrhythmias, pericarditis, and pericardial effusion (Takeda Pharmaceuticals, 2022). Pediatric incidence of cardiac AEs was 5.51%, with 2.06% experiencing ≥ grade 3 toxicity (Tables 1, 2; Figure 2D). Toxicities reported in pediatric patients included heart failure, left ventricular systolic dysfunction, and hypotension. In young pediatric patients, the overall incidence was 0.91% (Table 3). ALL and AML patients had a 6.82% incidence of cardiac toxicities. When ALL and AML were excluded, 1.1% of pediatric patients and no young pediatric patients experienced cardiac AEs (Tables 1, 3; Figure 2D). In summary, pediatric patients experienced a lower incidence of cardiac AEs than adults.

### Dose limiting toxicities (DLTs)

DLT occurred in 13% of adults treated with BTZ, with the most common reasons for discontinuation being PN (5%) and diarrhea (3%) (Takeda Pharmaceuticals, 2022). Pediatric incidence of DLT was comparable at 13.78% (Table 1; Figure 2E). Reasons for discontinuation, reduction, or delay in dose were often not reported. The most frequently specified DLT was GI toxicity (0.65%), thrombocytopenia (0.65%), or PN (0.65%). In young pediatric patients, the incidence of DLT was lower than adults at 10.66% (Table 3). ALL and AML patients had a 16.09% incidence of dose-limiting toxicities. When patients with ALL and AML were excluded, the incidence of DLT was even lower at 7.01% of all pediatric patients and 3.45% of young pediatric patients (Tables 1, 3; Figure 2E).

### Deaths

Deaths while on BTZ therapy were rare. Adult mortality rate during BTZ treatment (with or without other concurrent treatment) ranged from 0.17% to 4% (Takeda Pharmaceuticals, 2022; Bringhen et al., 2018). Pediatric mortality rate was 1.92% (Table 1; Figure 2E), with an incidence for young pediatric patients of 0.82% (Table 3). The most common cause of on-therapy mortality was infection. Infections were predominantly fungal (5/8) and occurred in the context of broad immunosuppression with co-therapy of combinations of vincristine, dexamethasone, pegylated L-asparaginase, doxorubicin, intrathecal methotrexate, and/or intrathecal triple chemotherapy (methotrexate, cytarabine, methylprednisolone or hydrocortisone) (Supplementary Table S4). Of the 12 pediatric deaths reported to be possibly related to BTZ therapy, 11 occurred in patients with ALL (Table 3; Figure 2E). One death (0.60%) occured after heart transplantation due to renal and pulmonary complications (Morrow et al., 2012).

### Toxicities when bortezomib was used as a single agent

Of the 64 articles used in this systematic review, 4 articles (2 clinical trials and 2 case series/reports) described the use of BTZ as a single agent (Supplementary Tables S1–S3). This cohort of patients had higher neurological toxicities (4.55%) and GI toxicities (31.82%) (Figure 2; Supplementary Table S7). Interestingly, they had similar incidence of toxicities relative to non-ALL or AML patients receiving multi-therapy.

### Certainty of evidence and risk of bias

A significant limitation of this systematic review is a paucity of blinded pediatric studies of BTZ. The majority of studies (48/64) included in this analysis were case reports or case series ≤10 patients and did not have a systematic framework for evaluating AEs. The risk of researcher influence or selection bias in cases chosen for publication was also possible.

## Discussion

Our systematic review demonstrates that the BTZ toxicity profile for pediatric patients differs from adults. Adult BTZ toxicities affect the nervous, cardiac and pulmonary, GI systems, and cause bone marrow suppression and infections. Pediatric patients had similar or higher incidence of bone marrow suppression and infection, but decreased incidence of neurological, cardiac, pulmonary, and GI AEs.

Evaluation of BTZ’s safety profile in pediatric patients is complicated by its use in conjunction with multiple chemotherapeutic agents and in immunosuppressed ALL, AML and organ transplantation patients. Patients meeting these criteria comprised approximately 50% of pediatric study patients (Table 1; Supplementary Tables S4–S6). Non-ALL or AML patients experienced lower incidence of PN, pulmonary toxicities, and infectious complications, and demonstrates that BTZ toxicity is likely exacerbated by co-morbidities (Table 1). Pediatric patients receiving BTZ as a single agent had toxicities similar to non-ALL or AML patients receiving multiple therapies.

PN is one of the most common and dose-limiting toxicity in adults treated with BTZ (Argyriou et al., 2008; Yamamoto and Egashira, 2021; Yang et al., 2024), affecting up to 38% of adult patients (Takeda Pharmaceuticals, 2022). Although poorly understood, it is believed that BTZ affects glial cells, causing axonal degeneration, impairing axonal transport, and inducing oxidative stress and inflammation in the peripheral nervous system (Yamamoto and Egashira, 2021; Yang et al., 2024). Pediatric patients treated with BTZ had a 4-fold lower incidence of PN when compared to adults (8.6%, vs. 38%, Table 1). When ALL pediatric patients were excluded, the incidence of PN was nearly 9-fold lower (4.4% Table 1). Moreover, PN in pediatric patients was generally low-grade and reversible. There was a higher incidence of PN in ALL and AML patients, compared to non-ALL or AML indications. When BTZ was used as a single agent, the PN incidence was as high as the ALL and AML patients (Supplementary Table S7). The majority of this cohort were treated for oncologic indications. This may suggest that oncologic patients inherently are more sensitive to neurotoxic effects of BTZ. A phase 1 clinical trial of ALL patients using BTZ and vincristine reported 83% of patients with grade 2 PN (Iguchi et al., 2017), while it was not reported in another clinical trial of ALL patients in which BTZ was used as a single agent (Horton et al., 2007). These data suggest that pediatric patients had more favorable risk profile of PN compared to adults. A recent paper by Joshi et al. found that pediatric ALL patients who experienced PN during vincristine treatment tolerated a switch to BTZ with equivocal efficacy and significantly less PN (Joshi et al., 2019). Our study confirmed that the AE profile of BTZ may be more favorable than vincristine for ALL patients.

Pediatric patients receiving BTZ also have low incidence of cardiopulmonary and GI toxicities. Adult cardiovascular toxicities were dose-dependent and included new or worsening heart failure and ischemic heart disease (Takeda Pharmaceuticals, 2022). Pediatric cardiac AEs were less common than adults and included hypotension, left ventricular dysfunction, and heart failure. GI toxicities for both adult and pediatric patients included nausea, vomiting, diarrhea and were lower in pediatric patients compared to adults. Incidence of adult pulmonary AEs were not reported in the drug insert (Takeda Pharmaceuticals, 2022); pediatric pulmonary AEs included hypoxia, respiratory failure, and ARDS.

Several factors may contribute to the higher incidence of cardiac and GI toxicities observed in adults relative to pediatric patients. Research in neurodegenerative diseases has found that the proteasomal degradation activity decreases with age (Rousseau and Bertolotti, 2018). This age-induced proteosome instability may contribute to the worsening of the AEs to PIs (Georgiopoulos et al., 2023), which may explain why pediatric patients experience less BTZ-induced neurological, cardiac and GI AEs. Additionally, adults are more likely to have pre-existing comorbidities that increase the risk of developing BTZ-induced AEs such as cardiotoxicity and neuropathy (Georgiopoulos et al., 2023). Neuropathy-inducing comorbidities such as diabetes mellitus are more common in adults and can increase the risk of BTZ-induced PN (Lanzani et al., 2008; Bruna et al., 2010). Neuropathy secondary to multiple myeloma has been shown to increase the likelihood of developing neuropathy following BTZ treatment. These data may explain why pediatric patients have lower incidence of PN from BTZ treatment (Meregalli et al., 2015). Similarly, cardiovascular risk factors, which have a higher prevalence in older patients, increase the risk of BTZ-induced cardiotoxicity (Kistler et al., 2017). Decline of the proteosome activity, differences in BTZ indication, and age-associated comorbidities all may contribute to the lower rate of neurological, cardiac and GI toxicities in pediatric patients.

Pediatric patients treated with BTZ experience high incidence of bone marrow suppression including anemia, neutropenia, and thrombocytopenia ranging from 17% to 50% (Table 1). When pediatric patients with ALL were excluded, the pediatric AE incidences were decreased to <30%, comparable to adults. In contrast, ALL and AML patients had rates of bone marrow suppression up to 80%, which puts them at higher risk for infection. ALL/recurrent ALL and AML patients in these published studies were on multi-agent regimens which confounds the contribution of BTZ in bone marrow suppression in this patient population (Table 1). Horton et al. concluded that while BTZ might have exacerbated thrombocytopenia, the rates were not different from historical controls of ALL patients without BTZ treatment (Horton et al., 2019). Pediatric leukopenia and lymphopenia incidence were comparable to adults (Robak et al., 2018) (Tables 1-3).

The most significant pediatric BTZ toxicity is infection (42.8%), which occurred in most deaths potentially attributable to BTZ (Table 1) (Horton et al., 2007; Messinger et al., 2010; Yeo et al., 2016; Bertaina et al., 2017; Iguchi et al., 2017; Kaspers et al., 2018; Hasegawa et al., 2019; Horton et al., 2019; Roy et al., 2019; August et al., 2020; Colunga-Pedraza et al., 2020; Ravichandran et al., 2021). Infection is a serious concern during treatment with BTZ and is especially common in patients with blood malignancies. A high rate of infection may be expected in heavily pretreated and immunosuppressed patient groups (Takeda Pharmaceuticals, 2022). The frequency and severity of infections observed in BTZ-treated pediatric ALL patients were comparable to those observed in studies of recurrent ALL patients receiving standard reinduction chemotherapy (Horton et al., 2019; August et al., 2020). These studies suggest that the risk of infection attributable specifically to BTZ may be over-estimated in ALL and AML patients concurrently treated with other chemotherapeutic agents. These results confirm ALL and AML patients are at increased infectious risks from their underlying primary disease and immunsuppressive effects of concurrent therapies in addition to BTZ. Infectious toxicities in pediatric patients treated with BTZ for non-ALL/or was much lower (5%) (Table 1). Nevertheless, bone marrow suppression remains the major toxicity that is experienced in non-ALL or AML pediatric patients.

This study had major shortcomings. The decision to include publications with toxicity data for patients ≤18 years of age disqualified many clinical trials from analysis. This decision was based on older teenager and young adults (18–21) having similar physiology to adults and thus experienced toxicities may not reflect true pediatric response to BTZ. For this same reason, we further stratified patients into young pediatric patients (≤8 years old) when data was available for analysis. Our data demonstrated that younger pediatric patients tolerated BTZ better than older pediatric patients. This is consistent with other reports that younger ALL pediatric patients (≤14 years) have decreased treatment-related toxicities compared to adolescents and young adults using pediatric protocols (Gupta et al., 2021; Janardan and Miller, 2023). The majority of source publications were smaller clinical studies (>10 patients) or case report/series (≤10 patients) and thus more anecdotal than well-controlled clinical trials. This uneven distribution of patient population may confound our findings that ALL and AML patients experience higher incidence of AEs. Nevertheless, this review endeavored to include pediatric patients undergoing BTZ treatment for both oncologic and non-oncologic indications with a goal to comprehensively review BTZ toxicities in pediatric patients. Even though there is a lack of well-controlled clinical trials for pediatric patients undergoing BTZ treatment (with or without concurrent therapies) for non-oncologic indications, the collective experience in the medical literature for this population should be reviewed and analyzed as BTZ is being used for a widening range of indications in the pediatric setting.

## Conclusion

Our systematic review demonstrates that BTZ is acceptable for use in pediatric patients. Pediatric patients with ALL or AML had higher risks for AEs and ≥grade 3 AEs. PN, GI, and pulmonary toxicities were lower in pediatric patients than adults. In contrast, bone marrow suppression and infection were the major AEs for pediatric patients and occurred at rates comparable to those in adults. Finally, dose-limiting toxicities were comparable to the adult experience (Blaney et al., 2004; Kaspers et al., 2018; August et al., 2020). Due to the high incidence of bone marrow suppression and infection in ALL and AML patients, antibiotic and anti-fungal prophylaxis while on BTZ therapy is recommended to minimize infection/sepsis risks. While infections occurred at a lower rate for non-ALL or AML patients, bone marrow suppression remained the main toxicity that is experienced. Therefore, antibiotic and anti-fungal prophylaxis should also be considered for non-ALL or AML patients on BTZ. As BTZ targets pathologic cells with abnormal protein accumulation, using doses lower than standard oncologic doses in non-malignant and non-transplantation pediatric patients may also provide efficacy while minimizing AEs.

## References

[B1] Adams J. (2004). The development of proteasome inhibitors as anticancer drugs. Cancer Cell 5, 417–421. 10.1016/s1535-6108(04)00120-5 15144949

[B2] Argyriou A. A. Iconomou G. Kalofonos H. P. (2008). Bortezomib-induced peripheral neuropathy in multiple myeloma: a comprehensive review of the literature. Blood 112, 1593–1599. 10.1182/blood-2008-04-149385 18574024

[B3] August K. J. Guest E. M. Lewing K. Hays J. A. Gamis A. S. (2020). Treatment of children with relapsed and refractory acute lymphoblastic leukemia with mitoxantrone, vincristine, pegaspargase, dexamethasone, and bortezomib. Pediatr. Blood Cancer 67, e28062. 10.1002/pbc.28062 31724803

[B4] Bertaina A. Vinti L. Strocchio L. Gaspari S. Caruso R. Algeri M. (2017). The combination of bortezomib with chemotherapy to treat relapsed/refractory acute lymphoblastic leukaemia of childhood. Br. J. Haematol. 176, 629–636. 10.1111/bjh.14505 28116786

[B5] Blaney S. M. Bernstein M. Neville K. Ginsberg J. Kitchen B. Horton T. (2004). Phase I study of the proteasome inhibitor bortezomib in pediatric patients with refractory solid tumors: a children's oncology group study (ADVL0015). J. Clin. Oncol. 22, 4804–4809. 10.1200/JCO.2004.12.185 15570082

[B6] Bringhen S. Offidani M. Palmieri S. Pisani F. Rizzi R. Spada S. (2018). Early mortality in myeloma patients treated with first-generation novel agents thalidomide, lenalidomide, bortezomib at diagnosis: a pooled analysis. Crit. Rev. Oncol. Hematol. 130, 27–35. 10.1016/j.critrevonc.2018.07.003 30196909

[B7] Bruna J. Udina E. Ale A. Vilches J. J. Vynckier A. Monbaliu J. (2010). Neurophysiological, histological and immunohistochemical characterization of bortezomib-induced neuropathy in mice. Exp. Neurol. 223, 599–608. 10.1016/j.expneurol.2010.02.006 20188093

[B8] Cao M. Luo X. Wu K. He X. (2021). Targeting lysosomes in human disease: from basic research to clinical applications. Signal Transduct. Target Ther. 6, 379. 10.1038/s41392-021-00778-y 34744168 PMC8572923

[B9] Chauhan D. Catley L. Li G. Podar K. Hideshima T. Velankar M. (2005). A novel orally active proteasome inhibitor induces apoptosis in multiple myeloma cells with mechanisms distinct from bortezomib. Cancer Cell 8, 407–419. 10.1016/j.ccr.2005.10.013 16286248

[B10] Chauhan D. Singh A. V. Aujay M. Kirk C. J. Bandi M. Ciccarelli B. (2010). A novel orally active proteasome inhibitor ONX 0912 triggers *in vitro* and *in vivo* cytotoxicity in multiple myeloma. Blood 116, 4906–4915. 10.1182/blood-2010-04-276626 20805366 PMC3321748

[B11] Ciechanover A. (2005). Proteolysis: from the lysosome to ubiquitin and the proteasome. Nat. Rev. Mol. Cell Biol. 6, 79–87. 10.1038/nrm1552 15688069

[B12] Colunga-Pedraza J. E. Gonzalez-Llano O. Gonzalez-Martinez C. E. Gomez-Almaguer D. Yanez-Reyes J. M. Jimenez-Antolinez V. (2020). Outpatient low toxic regimen with bortezomib in relapsed/refractory acute lymphoblastic leukemia in pediatrics and AYA patients: single-center Mexican experience. Pediatr. Blood Cancer 67, e28241. 10.1002/pbc.28241 32159276

[B13] Crawford L. J. Walker B. Irvine A. E. (2011). Proteasome inhibitors in cancer therapy. J. Cell Commun. Signal 5, 101–110. 10.1007/s12079-011-0121-7 21484190 PMC3088792

[B14] Crawford L. J. Windrum P. Magill L. Melo J. V. Mccallum L. Mcmullin M. F. (2009). Proteasome proteolytic profile is linked to Bcr-Abl expression. Exp. Hematol. 37, 357–366. 10.1016/j.exphem.2008.11.004 19157685

[B15] Cromm P. M. Crews C. M. (2017). The proteasome in modern drug discovery: second life of a highly valuable drug target. ACS Cent. Sci. 3, 830–838. 10.1021/acscentsci.7b00252 28852696 PMC5571462

[B16] Cvek B. Dvorak Z. (2008). The value of proteasome inhibition in cancer. Can the old drug, disulfiram, have a bright new future as a novel proteasome inhibitor? Drug Discov. Today 13, 716–722. 10.1016/j.drudis.2008.05.003 18579431

[B17] Delic J. Masdehors P. Omura S. Cosset J. M. Dumont J. Binet J. L. (1998). The proteasome inhibitor lactacystin induces apoptosis and sensitizes chemo- and radioresistant human chronic lymphocytic leukaemia lymphocytes to TNF-alpha-initiated apoptosis. Br. J. Cancer 77, 1103–1107. 10.1038/bjc.1998.183 9569046 PMC2150120

[B18] Georgiopoulos G. Makris N. Laina A. Theodorakakou F. Briasoulis A. Trougakos I. P. (2023). Cardiovascular toxicity of proteasome inhibitors: underlying mechanisms and Management Strmtegies. JACC CardioOncol 5, 1–21. 10.1016/j.jaccao.2022.12.005 36875897 PMC9982226

[B19] Gupta A. Damania R. C. Talati R. O'riordan M. A. Matloub Y. H. Ahuja S. P. (2021). Increased Toxicity Among Adolescents and Young Adults Compared with Children Hospitalized with Acute Lymphoblastic Leukemia at Children's Hospitals in the United States. J. Adolesc. Young Adult Oncol. 10, 645–653. 10.1089/jayao.2020.0154 33512257

[B20] Hasegawa D. Yoshimoto Y. Kimura S. Kumamoto T. Maeda N. Hara J. (2019). Bortezomib-containing therapy in Japanese children with relapsed acute lymphoblastic leukemia. Int. J. Hematol. 110, 627–634. 10.1007/s12185-019-02714-x 31401767

[B21] Horton T. M. Pati D. Plon S. E. Thompson P. A. Bomgaars L. R. Adamson P. C. (2007). A phase 1 study of the proteasome inhibitor bortezomib in pediatric patients with refractory leukemia: a Children's Oncology Group study. Clin. Cancer Res. 13, 1516–1522. 10.1158/1078-0432.CCR-06-2173 17332297

[B22] Horton T. M. Whitlock J. A. Lu X. O'brien M. M. Borowitz M. J. Devidas M. (2019). Bortezomib reinduction chemotherapy in high-risk ALL in first relapse: a report from the Children's Oncology Group. Br. J. Haematol. 186, 274–285. 10.1111/bjh.15919 30957229 PMC6606340

[B23] Iguchi A. Cho Y. Sugiyama M. Terashita Y. Ariga T. Hosoya Y. (2017). Bortezomib combined with standard induction chemotherapy in Japanese children with refractory acute lymphoblastic leukemia. Int. J. Hematol. 106, 291–298. 10.1007/s12185-017-2235-z 28401497

[B24] Jagannath S. Barlogie B. Berenson J. Siegel D. Irwin D. Richardson P. G. (2004). A phase 2 study of two doses of bortezomib in relapsed or refractory myeloma. Br. J. Haematol. 127, 165–172. 10.1111/j.1365-2141.2004.05188.x 15461622

[B25] Janardan S. K. Miller T. P. (2023). Adolescents and young adults (AYAs) vs pediatric patients: survival, risks, and barriers to enrollment. Hematol. Am. Soc. Hematol. Educ. Program 2023, 581–586. 10.1182/hematology.2023000507 PMC10727024 38066874

[B26] Joshi J. Tanner L. Gilchrist L. Bostrom B. (2019). Switching to Bortezomib may Improve Recovery From Severe Vincristine Neuropathy in Pediatric Acute Lymphoblastic Leukemia. J. Pediatr. Hematol. Oncol. 41, 457–462. 10.1097/MPH.0000000000001529 31233464

[B27] Kaspers G. J. L. Niewerth D. Wilhelm B. a.J. Scholte-Van Houtem P. Lopez-Yurda M. Berkhof J. (2018). An effective modestly intensive re-induction regimen with bortezomib in relapsed or refractory paediatric acute lymphoblastic leukaemia. Br. J. Haematol. 181, 523–527. 10.1111/bjh.15233 29676440

[B28] Kim H. Y. Moon J. Y. Ryu H. Choi Y. S. Song I. C. Lee H. J. (2015). Bortezomib inhibits the survival and proliferation of bone marrow stromal cells. Blood Res. 50, 87–96. 10.5045/br.2015.50.2.87 26157778 PMC4486164

[B29] Kistler K. D. Kalman J. Sahni G. Murphy B. Werther W. Rajangam K. (2017). Incidence and Risk of Cardiac Events in Patients With Previously Treated Multiple Myeloma Versus Matched Patients Without Multiple Myeloma: An Observational, Retrospective, Cohort Study. Clin. Lymphoma Myeloma Leuk. 17, 89–96. 10.1016/j.clml.2016.11.009 28025038

[B30] Lanzani F. Mattavelli L. Frigeni B. Rossini F. Cammarota S. Petro D. (2008). Role of a pre-existing neuropathy on the course of bortezomib-induced peripheral neurotoxicity. J. Peripher Nerv. Syst. 13, 267–274. 10.1111/j.1529-8027.2008.00192.x 19192066

[B31] Meregalli C. Carozzi V. A. Sala B. Chiorazzi A. Canta A. Oggioni N. (2015). Bortezomib-induced peripheral neurotoxicity in human multiple myeloma-bearing mice. J. Biol. Regul. Homeost. Agents 29, 115–124. 25864747

[B32] Messinger Y. Gaynon P. Raetz E. Hutchinson R. Dubois S. Glade-Bender J. (2010). Phase I study of bortezomib combined with chemotherapy in children with relapsed childhood acute lymphoblastic leukemia (ALL): a report from the therapeutic advances in childhood leukemia (TACL) consortium. Pediatr. Blood Cancer 55, 254–259. 10.1002/pbc.22456 20582937

[B33] Moreau P. Pylypenko H. Grosicki S. Karamanesht I. Leleu X. Grishunina M. (2011). Subcutaneous versus intravenous administration of bortezomib in patients with relapsed multiple myeloma: a randomised, phase 3, non-inferiority study. Lancet Oncol. 12, 431–440. 10.1016/S1470-2045(11)70081-X 21507715

[B34] Morrow W. R. Frazier E. A. Mahle W. T. Harville T. O. Pye S. E. Knecht K. R. (2012). Rapid reduction in donor-specific anti-human leukocyte antigen antibodies and reversal of antibody-mediated rejection with bortezomib in pediatric heart transplant patients. Transplantation 93, 319–324. 10.1097/TP.0b013e31823f7eea 22179403 PMC3730122

[B35] Murai K. Kowata S. Shimoyama T. Yashima-Abo A. Fujishima Y. Ito S. (2014). Bortezomib induces thrombocytopenia by the inhibition of proplatelet formation of megakaryocytes. Eur. J. Haematol. 93, 290–296. 10.1111/ejh.12342 24750292

[B36] Muz B. Ghazarian R. N. Ou M. Luderer M. J. Kusdono H. D. Azab A. K. (2016). Spotlight on ixazomib: potential in the treatment of multiple myeloma. Drug Des. Devel Ther. 10, 217–226. 10.2147/DDDT.S93602 PMC4714737 26811670

[B37] Nawrocki S. T. Carew J. S. Pino M. S. Highshaw R. A. Dunner K. Jr. Huang P. (2005). Bortezomib sensitizes pancreatic cancer cells to endoplasmic reticulum stress-mediated apoptosis. Cancer Res. 65, 11658–11666. 10.1158/0008-5472.CAN-05-2370 16357177

[B38] Perel G. Bliss J. Thomas C. M. (2016). Carfilzomib (Kyprolis): A Novel Proteasome Inhibitor for Relapsed And/or Refractory Multiple Myeloma. P T 41, 303–307. 27162470 PMC4849338

[B39] Piva R. Ruggeri B. Williams M. Costa G. Tamagno I. Ferrero D. (2008). CEP-18770: A novel, orally active proteasome inhibitor with a tumor-selective pharmacologic profile competitive with bortezomib. Blood 111, 2765–2775. 10.1182/blood-2007-07-100651 18057228

[B40] Potts B. C. Albitar M. X. Anderson K. C. Baritaki S. Berkers C. Bonavida B. (2011). Marizomib, a proteasome inhibitor for all seasons: preclinical profile and a framework for clinical trials. Curr. Cancer Drug Targets 11, 254–284. 10.2174/156800911794519716 21247382 PMC3712795

[B41] Raedler L. (2015). Velcade (Bortezomib) Receives 2 New FDA Indications: For Retreatment of Patients with Multiple Myeloma and for First-Line Treatment of Patients with Mantle-Cell Lymphoma. Am. Health Drug Benefits 8, 135–140. 26629279 PMC4665054

[B42] Ravichandran N. Uppuluri R. Swaminathan V. V. Patel S. Ramanan K. M. Jayakumar I. (2021). FLAG With Bortezomib in Childhood Relapsed/Refractory Leukemia: Remission Induction With Limited Toxicity in the Era of Multidrug-resistant Bacteria. J. Pediatr. Hematol. Oncol. 43, e212–e214. 10.1097/mph.0000000000001644 31688621

[B43] Richardson P. G. Barlogie B. Berenson J. Singhal S. Jagannath S. Irwin D. (2003a). A phase 2 study of bortezomib in relapsed, refractory myeloma. N. Engl. J. Med. 348, 2609–2617. 10.1056/NEJMoa030288 12826635

[B44] Richardson P. G. Hideshima T. Anderson K. C. (2003b). Bortezomib (PS-341): a novel, first-in-class proteasome inhibitor for the treatment of multiple myeloma and other cancers. Cancer Control 10, 361–369. 10.1177/107327480301000502 14581890

[B45] Richardson P. G. Sonneveld P. Schuster M. W. Irwin D. Stadtmauer E. A. Facon T. (2005). Bortezomib or high-dose dexamethasone for relapsed multiple myeloma. N. Engl. J. Med. 352, 2487–2498. 10.1056/nejmoa043445 15958804

[B46] Robak T. Jin J. Pylypenko H. Verhoef G. Siritanaratkul N. Drach J. (2018). Frontline bortezomib, rituximab, cyclophosphamide, doxorubicin, and prednisone (VR-CAP) versus rituximab, cyclophosphamide, doxorubicin, vincristine, and prednisone (R-CHOP) in transplantation-ineligible patients with newly diagnosed mantle cell lymphoma: final overall survival results of a randomised, open-label, phase 3 study. Lancet Oncol. 19, 1449–1458. 10.1016/s1470-2045(18)30685-5 30348538

[B47] Rousseau A. Bertolotti A. (2018). Regulation of proteasome assembly and activity in health and disease. Nat. Rev. Mol. Cell Biol. 19, 697–712. 10.1038/s41580-018-0040-z 30065390

[B48] Roy P. Islam R. Saha D. Gogoi M. Kumar Mishra D. Arora N. (2019). Efficacy and safety of a bortezomib and reduced-intensity cytarabine-based protocol, TMC ALLR1, for relapsed childhood ALL in India. Br. J. Haematol. 186, 861–865. 10.1111/bjh.16005 31168836 PMC6785345

[B49] Saglam B. Kalyon H. Ozbalak M. Ornek S. Keske S. Tabak L. (2020). Bortezomib induced pulmonary toxicity: a case report and review of the literature. Am. J. Blood Res. 10, 407–415. 33489450 PMC7811899

[B50] San Miguel J. F. Schlag R. Khuageva N. K. Dimopoulos M. A. Shpilberg O. Kropff M. (2008). Bortezomib plus melphalan and prednisone for initial treatment of multiple myeloma. N. Engl. J. Med. 359, 906–917. 10.1056/nejmoa0801479 18753647

[B51] Takeda Pharmaceuticals (2022). Velcade_Drug_Insert Velcade (bortezomib). Cambridge, MA: Takeda Pharmaceuticals.

[B52] Teicher B. A. Tomaszewski J. E. (2015). Proteasome inhibitors. Biochem. Pharmacol. 96, 1–9. 10.1016/j.bcp.2015.04.008 25935605

[B53] Vogl D. T. Martin T. G. Vij R. Hari P. Mikhael J. R. Siegel D. (2017). Phase I/II study of the novel proteasome inhibitor delanzomib (CEP-18770) for relapsed and refractory multiple myeloma. Leuk. Lymphoma 58, 1872–1879. 10.1080/10428194.2016.1263842 28140719

[B54] Wang J. Fang Y. Fan R. A. Kirk C. J. (2021). Proteasome Inhibitors and Their Pharmacokinetics, Pharmacodynamics, and Metabolism. Int. J. Mol. Sci. 22, 11595. 10.3390/ijms222111595 34769030 PMC8583966

[B55] Yamamoto S. Egashira N. (2021). Pathological Mechanisms of Bortezomib-Induced Peripheral Neuropathy. Int. J. Mol. Sci. 22, 888. 10.3390/ijms22020888 33477371 PMC7830235

[B56] Yang Y. Zhao B. Lan H. Sun J. Wei G. (2024). Bortezomib-induced peripheral neuropathy: Clinical features, molecular basis, and therapeutic approach. Crit. Rev. Oncol. Hematol. 197, 104353. 10.1016/j.critrevonc.2024.104353 38615869

[B57] Yeo K. K. Gaynon P. S. Fu C. H. Wayne A. S. Sun W. (2016). Bortezomib, Dexamethasone, Mitoxantrone, and Vinorelbine (BDMV): An Active Reinduction Regimen for Children With Relapsed Acute Lymphoblastic Leukemia and Asparaginase Intolerance. J. Pediatr. Hematol. Oncol. 38, 345–349. 10.1097/MPH.0000000000000560 27352191 PMC7451259

