# Supplementary material for: Clinical use and toxicities of bortezomib in pediatric patients: a systematic review
Source: Front Pharmacol. 2025 Aug 6;16:1661493. doi: 10.3389/fphar.2025.1661493 (PMC12364856; doi:10.3389/fphar.2025.1661493)
Supplement: Supplementary file 1 [file Table1.docx]

| **Supplemental Table 1. Clinical trials of pediatric patients treated with BTZ** | | | | | | | |
| --- | --- | --- | --- | --- | --- | --- | --- |
| **Study/Study Name** | **Study Design** | **Indication** | **Number of patients evaluable for toxicity** | **Patient Age** | **Co-treatment** | **Route of administration** | **Dose and dose schedule** |
| Blaney (2004)  COG ADVL0015 | Phase 1 clinical trial | Recurrent solid tumors^1^ | 15 | 5-17, median: 11 | None | IV | 1.2 or 1.6 mg/m^2^, 4 doses for 1 cycle (9), 2 cycles (1), or 6 cycles (1) |
| Horton (2007)  COGADVL0317 | Phase 1 clinical trial | ALL (9), AML (3) | 5 | 1-18, median: 11 | None | Not reported | 1.3 or 1.7 mg/m^2^, 4 doses for 1 cycle |
| Messinger (2010)  TACLPhase 1: T2005-003 | Phase 1 clinical trial | Recurrent ALL (9 B-ALL, 1 T-ALL) | 10 | 3.3-16.4, median: 9.8 | V, X, L, D, IT-M, IT-T | Not reported | 1 mg/m^2^ or 1.3 mg/m^2^, 4 doses for 1 cycle |
| Muscal (2013)  COG ADVL0916 | Phase 1 clinical trial | Recurrent solid tumors^2^ | 17 | 1.1 - 20.1, median: 12.6 | Vorinostat | IV | 1.3 mg/m^2^, 4 doses for 1-4 cycles |
| Mody (2017) | Phase 1 clinical trial | Recurrent high-risk neuroblastoma | 18 | 1 -21, median: 5 | Irinotecan | IV | 1.2 mg/m^2^, 4 doses for a mean of 8.2 cycles (range 1-48, median: 2) |
| Iguchi (2017) | Phase 1 clinical trial | Recurrent B-ALL | 6 | 10 - 16, mean: 13.5 | Regimen A: V, X, L, D    Regimen B: V, X, L, Mi    IT-T if CNS involvement | Not reported | 1.3 mg/m^2^, 4 doses for 1 cycle |
| Kaspers (2018) | Phase 1 clinical trial | Recurrent ALL | 29 | 1.0 - 17.5, median: 9.8 | V, X | Not reported | 1.3 mg/m^2^, 4 doses for 1 cycle (10), 2 cycles (13), 3 cycles (4), 4 cycles (2) |
| Hasegawa (2019) | Phase 1 clinical trial | Recurrent ALL (2 B-ALL, 1 T-ALL) | 3 | 5.8 - 7.3, median: 6.7 | V, P, L, daunorubicin, cyclophosphamide, IT-T | IV | 1.3 mg/m^2^, 4 doses for 1 cycle |
| Roy (2019) | Phase 2 clinical trial | Recurrent ALL | 25 | 2.1 - 17.5, median: 7.9 | X, L, M | IV | 1.3 mg/m^2^, 4 doses for 1 cycle |
| August (2020) | Phase 1 clinical trial | recurrent ALL (9 B-ALL, 1 T-ALL) | 10 | 0.9 - 18.5, mean: 11.2 | V, X, L, Mi, IT-M | IV | 1.3 mg/m^2^, 4 doses for 1 cycle |
| Aplenc (2020)  COG AAML1031 | Phase 3 clinical trial | Newly diagnosed AML | 287 | 0 - 29.5, median: 9.2  Included patients aged 0-15 | Cytarabine, daunorubicin, etoposide | Not reported | 1.3 mg/m^2^, 3 doses for 3-4 cycles |
| ^1^Optic glioma (1), osteosarcoma (2), hepatoblastoma (1), neuroblastoma (2), adenocarcinoma (1), Wilms’ tumor (2), rhabdomyosarcoma (2)  ^2^Malignant glioma (6), peripheral nerve sheath tumor (1), medulloblastoma (1), neuroblastoma (3), extrarenal rhabdoid tumor (2), Ewing sarcoma (2), rhabdomyosarcoma (1), hepatoblastoma (1), osteosarcoma (1), Wilms tumor (1), embryonal sarcoma (1), carcinoma (1), epithelioid sarcoma (1), retinoblastoma (1)    Vincristine (V), dexamethasone (X), prednisone (P), pegylated L-asparaginase (L), doxorubicin (D), methotrexate (M), intrathecal methotrexate (IT-M), intrathecal triple chemotherapy (methotrexate, cytarabine, methylprednisolone or hydrocortisone) (IT-T), intrathecal cytarabine (IT-C), mitoxantrone (Mi) | | | | | | | |

| **Supplemental Table 2. Clinical studies of pediatric patients treated with BTZ** | | | | | | | |
| --- | --- | --- | --- | --- | --- | --- | --- |
| **Study** | **Study Design** | **Indication** | **Number of patients evaluable for toxicity** | **Patient Age** | **Co-treatment** | **Route of administration** | **Dose and dose schedule** |
| Bertaina (2017) | Prospective | Recurrent ALL (B-ALL (30), T-ALL (7)) | 37 | 2.6-21, median: 10.6    (patients >18 excluded) | V, X, L, D, IT-M; IT-T if CNS involvement | IV | 1.3 mg/m^2^, 4 doses for 1 cycle |
| Kizilbash (2017) | Prospective | AMR renal transplant | 33 | 1-20; median: 12 | R (78% of patients), IVIG (90%), PEX (78%) | Not reported | 1.3 mg/m^2^, 3 or fewer doses (3), 4 doses (21), 5 doses (2), 8 doses (7) |
| Colunga-Pedraza (2020) | Retrospective | Recurrent ALL | 13 (2 patients >18 excluded) | 2-35, median: 13  (patients >18 excluded) | V, X, L, D, IT-T | SQ | 1.3 mg/m^2^, 4 doses for 1 cycle |
| Ravichandran (2021) | Retrospective | Recurrent ALL or AML (ALL (9), AML (4)) | 13 | 5-16, mean: 11 | FLAG (fludarabine, cytosine arabinoside, G-CSF) | IV | 1.3 mg/m^2^, 4 doses for 1 cycle |
| Miyagawa (2023) | Prospective | Recurrent ALL | 16 | 3-17 | Prednisolone, cyclophosphamide, daunorubicin, L, IT-T, V | IV | 1.3 mg/m^2^, 4 doses for 1 cycle |
| Vincristine (V), dexamethasone (X), pegylated L-asparaginase (L), doxorubicin (D), intrathecal methotrexate (IT-M), intrathecal triple chemotherapy (methotrexate, cytarabine, methylprednisolone or hydrocortisone) (IT-T), rituximab (R). | | | | | | | |

| **Supplemental Table 3. Case reports and case series of pediatric patients treated with BTZ** | | | | | | | |
| --- | --- | --- | --- | --- | --- | --- | --- |
| **Category** | **Study** | **Indication** | **Number of patients evaluable for toxicity** | **Patient Age** | **Co-treatment** | **Route of administration** | **Dose and dose schedule** |
| Transplant | Morrow (2012) | AMR cardiac transplant | 4 | 13 mo-5 yrs | R, PEX | IV | 1.3 mg/m^2^, 4 doses for 1 cycle (3) or 2 cycles (1) |
|  | Ryckewaert (2013) | AMR renal transplant | 1 | 17.5 | Methylprednisolone, P, IVIG, PEX | Not reported | 1.3 mg/m^2^, 4 doses for 2 cycles |
|  | Westphal (2013) | AMR renal transplant | 1 | 3.5 | IVIG, PEX | Not reported | 1.3 mg/m^2^, 4 doses for 1 cycle |
|  | Twombley (2013) | AMR renal transplant | 2 | 8.3, 16.8 | IVIG, PEX | Not reported | 1.3 mg/m^2^, 4 doses for 1 cycle |
|  | Zinn (2014) | AMR cardiac transplant | 3 | 4-15 | R. methylprednisolone, PEX | Not reported | 1.3 mg/m^2^, 4 doses for 1-3 cycles |
|  | Claes (2014) | AMR renal transplant | 1 | 12 | PEX | Not reported | 1.3 mg/m^2^, 4 doses for 1 cycle |
|  | Nguyen (2014) | AMR renal transplant | 4 | 3-19 | Methylprednisolone | IV | 1.3 mg/m^2^, 4 doses for 1 cycle |
|  | May (2014) | HLA desensitization prior to cardiac transplant | 1 | 9 | R, PEX | Not reported | 1.3 mg/m^2^, 5 doses for 1 cycle |
|  | Preston (2014) | Desensitization prior to lung transplant | 1 | 8 | Methylprednisone, R, IVIG, PEX | Not reported | Not reported |
|  | Roberti (2015) | AMR renal transplant | 2 | 17-18 | Methylprednisolone | IV | 1.3 mg/m^2^, 4 doses for 1 cycle |
|  | Hayes Jr. (2016) | AMR lung transplant | 1 | 15 mo | None | IV | 1.3 mg/m^2^, 4 doses for 1 cycle |
|  | Pearl (2016) | AMR renal transplant | 7 | 5-19 | R, methylprednisolone, IVIG, acetaminophen, diphenhydramine, PEX | Not reported | 1.3 mg/m^2^, 4 doses for 1-3 cycles |
|  | Pirojsakul (2016) | HLA desensitization prior to renal transplantation | 4 | 5-18 | R, IVIG | Not reported | 1.3 mg/m^2^, 4 doses for 1 cycle |
|  | Tasaki (2019) | AMR/PCAR renal transplant | 3 | 4-21 | R, IVIG, tacrolimus, cyclosporine A, methylprednisolone, MMF, rabbit antithymocyte globulin, PEX | Not reported | 1.3 mg/m^2^, 4 doses for 1 cycle |
|  | Sanai (2023) | GVHD prophylaxis post HSCT | 1 | 9 mo | Cyclophosphamide, cyclosporine, MMF | Not reported | 1.3 mg/m^2^, 2 doses post-transplant |
| Autoimmune cytopenias | Khandelwal (2014) | Refractory autoimmunity^1^ | 7 | 1.3-17.8 included (1 patient age 25 excluded) | R, PEX | IV (6 patients), SQ (1 patient) | 1.3 mg/m^2^, 4 doses for 1 cycle (7) or 2 cycles (2) |
|  | Waespe (2014) | AIC after HSCT for ALL | 1 | 1 | V, prednisolone, MMF, cyclosporine A, IVIG | Not reported | 1.3 mg/m^2^, 6 doses for 1 cycle |
|  | Mehta (2014) | AIHA after HSCT for SCID | 1 | 4 mo | R | IV | 1.3 mg/m^2^, 4 doses for 4 cycles |
|  | Van Balen (2014) | Refractory TTP | 1 | 16 | R, P, PEX | Not reported | 1.3 mg/m^2^, 4 doses for 1 cycle |
|  | Kruizinga (2018) | AIC after HSCT | 7 | 1-15 | P, IVIG | Not reported | 1.3 mg/m^2^, once weekly for 1-4 weeks |
|  | Knops (2020) | AIHA after intestinal transplant | 1 | 4 | High-dose corticosteroids | Not reported | 1.3 mg/m^2^, 4 doses for 1 cycle + 2 additional doses |
|  | Knight (2020) | Refractory Evans syndrome | 1 | 15 | P, MMF, IVIG | Not reported | 1.3 mg/m^2^, 5 doses for 1 cycle |
|  | Beydoun (2020) | Refractory Evans syndrome | 3 | 2-15 | Patient 1: None  Patient 2: P  Patient 3: X, romiplastim | Not reported | 1.3 mg/m^2^, 5 doses for 1 cycle (1).  1.3 mg/m^2^, 4 doses for 1 cycle (2) |
|  | Maschan (2020) | Refractory TTP | 2 | 5, 12 | PEX | IV | 1.3 mg/m^2^, 4 doses for 1-2 cycles |
|  | Azapagasi (2021) | Refractory TTP | 1 | 16 | N-acetylcysteine, PEX | Not reported | 1.0 mg/m^2^, 4 doses for 1 cycle |
|  | Conti (2022) | Refractory Evans syndrome | 1 | 14 | IVIG, P, sirolimus, eltrombopag, romiplostim, R | Not reported | 1.3 mg/m^2^, 4 doses for 1 cycle |
|  | Wali (2023) | Refractory TTP | 1 | 18 | R, Caplacizumab, P, PEX | Not reported | 1.3 mg/m^2^, 4 doses for 1 cycle |
| Anti-NMDA receptor encephalitis | Cordani (2019) | Refractory anti-NMDA receptor encephalitis | 1 | 8 | Sirolimus, lacosamide | IV | 1.3 mg/m^2^, 4 doses for 2 cycles |
|  | Turnbull (2020) | Refractory anti-NMDA receptor encephalitis | 1 | 18 | R | SQ | 1.3 mg/m^2^, 4 doses for 1 cycle |
|  | Simmons (2021) | Refractory anti-NMDA receptor encephalitis | 1 | 5 | X, P | IV, SQ | 1.3 mg/m^2^, 4 doses IV for 5 cycles, SQ for 6^th^ cycle |
|  | Govil-Dalela (2021) | Refractory anti-NMDA receptor encephalitis | 1 | 14 | None | Not reported | Dose not reported, 1 cycle |
| ALL | Hirabayashi (2014) | recurrent ALL | 1 | 18 | P, L, V | Not reported | 1.3 mg/m^2^, 12 doses for 1 cycle |
|  | Lo Nigro (2019) | recurrent B-ALL | 2 | 2 | V, X, L, R | IV | 1.3 mg/m^2^, 4 doses for 1 cycle |
|  | Wang (2020) | recurrent B-ALL | 1 | 15 | Vindesine, Idarubicin, D, L | Not reported | 1.0 mg/m^2^, 2 doses for 4 cycles |
|  | Ceolin (2022) | recurrent T-ALL | 1 | 12 | V, X, L, Mi | Not reported | 1.3 mg/m^2^, 4 doses for 1 cycle |
|  | Wang (2023) | recurrent B-ALL with TP53c.C275T mutation | 1 | 2 | V, cytarabine, fludarabine | Not reported | 1.0 mg/m^2^, 2 doses for 3 cycles |
| Solid Tumor | Carden (2017) | Metastatic renal medullary carcinoma | 2 | 10, 14 | Cisplatin, carboplatin, paclitaxel, gemcitabine | Not reported | 1 mg/m^2^, 3 doses every 3 weeks for 12 months then 1 mg/m^2^, 2 doses/week for 12 months (1) OR  1 mg/m^2^, 3 doses for 8 cycles then 1.5 mg/m^2^, 4 doses + 1.3 mg/m^2^, 1 dose (1) |
|  | Ryan (2021) | Renal medullary carcinoma | 1 | 14 (two patients >18 excluded) | Cisplatin, D, carboplatin, paclitaxel, gemcitabine | Not reported | 1.3 mg/m^2^, 3 dose for 4 cycles |
|  | Dalela (2023) | Renal medullary carcinoma | 1 | 13 | Cisplatin, D, carboplatin, paclitaxel, gemcitabine | Not reported | 1.3 mg/m^2^, 3 dose for 10 cycles |
| Other | Banugaria (2013) | High sustained antibody titers from ERT in Pompe disease | 3 | 4-6 | R, M, IVIG, cyclophosphamide, alglucosidase alpha | IV | 1.3 mg/m^2^, 4 doses for 3-6 cycles |
|  | Plant (2013) | Plasmacytoma-like post-transplant lymphoproliferative disorder | 2 | 2, 11 | D | Not reported | 1.6 mg/m^2^, 1 dose on weeks 1 and 2 for six 3-week cycles or 1.6 mg/m^2^, 1 dose for 3 weeks |
|  | Kim (2015) | Mucopolysaccharidosis type II | 1 | 6 | Ofatumumab, M, IVIG, idursulfase, D | IV | 1.3 mg/m^2^ weekly or biweekly in 4 week cycles for 32 weeks. |
|  | Kulkarni (2017) | Solitary plasmacytoma of bone | 1 | 12 | Lenalidomide, D | Not reported | Not reported |
|  | Torrealba (2018) | Proliferative glomerulonephritis with monoclonal immunoglobulin G deposits | 1 | 17 | Daratumumab, D | Not reported | Not reported |
|  | Epperly (2018) | Plasma cell myeloma | 1 | 9 | Lenalidomide, enoxaparin, zoledronic acid, D | Not reported | Not reported. 5 cycles of 21 days |
|  | Wang (2021) | Multiple myeloma | 1 | 14 | Cyclophosphamide, D | Not reported | 1.3 mg/m^2^, 4 doses for 5 cycles |
|  | Sasaki (2023) | Membranous nephropathy secondary to ERT in Pompe disease | 1 | 14 | R | SQ | 1.3 mg/m^2^, 4 doses for 2 cycles |
|  | Desai (2024) | High sustained antibody titers from ERT in Pompe disease | 2 | 9 mo, 2 yrs | Immune-tolerance induction M, R, IVIG | Not reported | Not reported, 4 doses for 4 cycles (1).  Not reported, 4 doses for 2 cycles (1). |
| ^1^AML (1), combined immune deficiency (2), SCID (2), Evan’s syndrome (1), unspecified immune deficiency (1)  Antibody-mediated rejection (AMR), rituximab (R), plasma exchange (PEX), vincristine (V), dexamethasone (X), prednisone (P), pegylated L-asparaginase (L), doxorubicin (D), methotrexate (M), mitoxantrone (Mi), mycophenolate mofetil (MMF), plasma cell-rich acute rejection (PCAR), human leukocyte antigen (HLA), autoimmune cytopenia (AIC), hematopoietic stem cell transplant (HSCT), acute lymphoblastic leukemia (ALL), severe combined immunodeficiency (SCID), autoimmune hemolytic anemia (AIHA), thrombotic thrombocytopenic purpura (TTP), enzyme replacement therapy (ERT). | | | | | | | |

| **Supplemental Table 4. Toxicities in clinical trials of pediatric patients treated with BTZ** | | | | | | | | | |
| --- | --- | --- | --- | --- | --- | --- | --- | --- | --- |
| **Study** | **Indication** | **# of evaluable patients** | **Neurological**  **(# of patients)** | **Hematological/Bone Marrow**  **(# of patients)** | **Infections**  **(# of patients)** | **Respiratory**  **(# of patients)** | **Gastrointestinal**  **(# of patients)** | **Cardiac**  **(# of patients)** | **DLTs**  **(# of patients)** |
| Blaney (2004) | Recurrent solid tumors^1^ | 15 | Peripheral neuropathy grade 1 (1) | Leukopenia: grade 2 (1)  Neutropenia: grade 2 (1), grade 3 (2), grade 4 (1)  Anemia: grade 2 (4), grade 3 (2)  Thrombocytopenia: grade 3 (3) | None | Not reported | Nausea/vomiting: grade 2 (2)  Diarrhea: grade 2 (1)  Abdominal pain: grade 2 (1) | Postural hypotension (1) | 2 patients with dose-limiting thrombocytopenia at 1.6 mg/m^2^ dose |
| Horton (2007) | ALL (9), AML (3) | 5 | CN XII/ confusion: grade 3 (1). | Not reported | Bacteremia (1), pneumonia (1) | None | Nausea: grade 3 (2)  Vomiting: grade 3 (3)  Anorexia: grade 2 (1), grade 3 (1)  GI bleed:  Grade 3 (1)    All AEs in 3 patients | Hypotension: grade 4 (1) | Grade 3 confusion/CN XII palsy (1), Grade 4 hypotension and febrile neutropenia (1).  Death from hypotension/ARDS  All at 1.7 mg/m^2^ dose |
| Messinger (2010) | Recurrent ALL (9 B-ALL, 1 T-ALL) | 10 | Peripheral neuropathy: grade 1 (1), grade 2 (1), transient.  Syncope: grade 1(1) | Anemia: grade 3 (6), grade 4 (1)  Neutropenia: grade 4 (7)  Febrile neutropenia: grade 3 (4)  Lymphopenia: grade 4 (1)  Leukopenia: grade 3 (1), grade 4 (8)  Thrombocytopenia: grade 4 (8). | Implant site infection: grade 3 (1)  Bacteriemia: bacterial (3), fungal (1). | None | Abdominal pain: grade 3 (1)  Typhlitis: grade 3 (1) | Hypotension: grade 3 (1) | Hypophosphatemia and rhabdomyolysis after one 1.3 mg/m^2^ dose (1)  Death from fungal infection (1) |
| Muscal (2013) | Recurrent solid tumors^2^ | 17 | Grade 2 confusion (1)  Peripheral neuropathy: Grade 2, resolved within two weeks of onset (1) or progressed to grade 4 (1). | Anemia: grade 2 (2), grade 3 (4)  Neutropenia: grade 2 (8), grade 3 (1)  Lymphopenia: grade 2 (4), grade 3 (5)  Leukopenia: grade 2 (4), grade 3 (1)  Thrombocytopenia: grade 2 (3), grade 3 (5), grade 4 (1) | None | Dyspnea and cough: grade 2 (1) | Abdominal pain grade 2 (1)  Anorexia: grade 2(1), grade 3(1)  Diarrhea: grade 2 (1)  Nausea: grade 2 (4), grade 3 (2)  Vomiting: grade 2 (6), grade 3 (2) | Not reported | 2 patients due to peripheral neuropathy. After fourth dose BTZ PN progressed from grade 2 to grade 4 (1) or repeat grade 2 PN during cycle 2 (1). |
| Mody (2017) | Recurrent high-risk neuroblastoma | 18 | Peripheral neuropathy: grades 1-2 (1), grade 3 (1). | Anemia: grades 1-2 (17), grade 3 (1)  Neutropenia: grades 1-2 (8), grade 3 (6), grade 4 (4)  Leukopenia: grades 1-2 (12), grade 3 (3), grade 4 (3)  Febrile Neutropenia: grade 3 (1)  Thrombocytopenia: grades 1-2 (12), grade 3 (3), grade 4 (3) | None | Not reported | Nausea/vomiting: grades 1-2 (17), grade 3 (1)  Diarrhea: grades 1-2 (12), grade 3 (2), grade 4 (1) | Not reported | 2 patients, attributable to high dose irinotecan/ Grade 4 thrombocytopenia (1) and grade 3 irritability (1) |
| Iguchi (2017) | Recurrent B-ALL | 6 | Peripheral neuropathy: grade 2 (5)  One patient with prolonged peripheral neuropathy at 2 year follow-up | Pancytopenia: grade 4 (6) | Bacterial pneumonia (1) Bacteremia (2) | None | None | Not reported | None |
| Kaspers (2018) | Recurrent ALL | 29 | Peripheral neuropathy: grade 3 (1), grade 4 (1)  Psychosis: grade 3 (1)  Depressed level of consciousness: grade 3 (1) | Anemia: grade 3 (2), grade 4 (16)  Neutropenia: grade 3 (5), grade 4 (17)  Leukopenia: grade 3 (7), grade 4 (16)  Thrombocytopenia: grade 3 (7), grade 4 (19) | Fungal: grade 3 (1)  Pulmonary: grade 3: (1)  Skin infection: grade 3 (1)  Sepsis: grade 3: (1) | Hypoxia: grade 3 (2) | Nausea: grade 3 (2)  Ileus: grade 4 (1) | Not reported | Cellulitis (1), febrile neutropenia (1)  Deaths from multisystem organ failure (1) and no specified cause (1) |
| August (2019) | Recurrent ALL (9 B-ALL, 1 T-ALL) | 10 | None | Neutropenia: grade 4 (10)  Thrombocytopenia: grade 3 and above (10) | Grade 3 (3), grade 4 (2), grade 5 (1)  (MRSA sepsis, HHV6 and CMV pneumonitis, fungal infection, bacteremia) | None | Not reported | Not reported | Death from fungal infection (1) |
| Roy (2019) | Recurrent ALL | 25 | None | Not reported | Grade 3 (17), grade 5 (2) | None | Mucositis: no grade (1) | Not reported | Death due to sepsis (2) |
| Hasegawa (2020) | Recurrent ALL (2 B-ALL, 1 T-ALL) | 3 | None | Anemia: grade 3 (3)  Neutropenia: grade 4 (3)  Febrile neutropenia: grade 3 (1)  Leukopenia: grade 4 (3)  Lymphopenia: grade 4 (3)  Thrombocytopenia: grade 4 (3) | No life-threatening infections | None | None | Not reported | None |
| Aplenc (2020) | Newly diagnosed AML | 287 | Peripheral neuropathy: grade unspecified (21)  Seizure (3) | Not reported | Microbiologically determined sterile site infections:  bacterial (193), fungal (9 | Hypoxia (47)  ARDS (7)  Respiratory failure (13) | Not reported | Cardiac LVSD (17)  EF decreased (19)  Heart Failure (15) | Unspecified (65) |
| Bortezomib (BTZ), peripheral neuropathy (PN), dose-limiting toxicities (DLTs), relapsed/refractory (r/r), acute myeloid leukemia (AML), acute lymphoblastic leukemia (ALL), pneumonia (PNA), bortezomib (BTZ), methicillin-resistant *Staphylococcus aureus* (MRSA), human herpesvirus 6 (HHV6), cytomegalovirus (CMV), Acute respiratory distress syndrome (ARDS), left ventricular systolic dysfunction (LVSD). | | | | | | | | | |

| **Supplemental Table 5. Toxicities in clinical studies of pediatric patients treated with BTZ** | | | | | | | | | |
| --- | --- | --- | --- | --- | --- | --- | --- | --- | --- |
| **Study** | **Indication** | **# of evaluable patients** | **Neurological**  **(# of patients)** | **Hematological/Bone Marrow**  **(# of patients)** | **Infections**  **(# of patients)** | **Respiratory**  **(# of patients)** | **Gastrointestinal**  **(# of patients)** | **Cardiac**  **(# of patients)** | **DLTs**  **(# of patients)** |
| Bertaina (2017) | Recurrent ALL (30 B-ALL, 7 T-ALL) | 37 | Peripheral neuropathy: grade 3 (4), grade 4 (1).  Central ataxia: grade 3 (1).  Completely resolved in all patients using gabapentin, vitamins B1 and B6, and neurophysiotherapy | Neutropenia: grade 3 (17), grade 4 (13)  Thrombocytopenia: grade 3 (16), grade 4 (13) | Fungal sepsis: grade 4 (3), grade 5 (3) | None | None | Not reported | Death due to fungal sepsis (3) |
| Kizilbash (2017) | AMR renal transplant | 33 | Headache (2) and Seizure (1) | Anemia: grade unspecified (2)  Neutropenia: grade unspecified (3)  Febrile neutropenia: grade unspecified (1).  Thrombocytopenia: grade unspecified (6) | None | Not reported | Nausea/vomiting/diarrhea: grade unspecified (5) | Not reported | None |
| Colunga-Pedraza (2020) | Recurrent ALL | 13 (2 patients >18 excluded) | Peripheral neuropathy: grade 1 (1), grade 2 (2). | Not reported | Herpes zoster (1), reactivation of pulmonary aspergillosis (1) | Not reported | Unspecified: grade 2 (1)  Pancreatitis: unspecified grade (1) | Not reported | Received only 3 doses due to moderate pancreatitis (1), received only 2 doses due to grade 2 GI toxicity (1) |
| Ravichandran (2021) | Recurrent ALL or AML (9 ALL, 4 AML) | 13 | Grade unspecified (3)  No additional cases with concurrent dexamethasone administration | Febrile neutropenia (13) | Culture positive sepsis  (5) | Not reported | None | Not reported | None |
| Miyagawa (2023) | Recurrent ALL | 16 | Peripheral neuropathy: grade 1 (2) | Anemia: grade 2 (5), grade 3 (1), grade 4 (2)  Neutropenia: grade 4 (11)  Febrile neutropenia: grade 3 (3)  Lymphopenia: grade 3 (3), grade 4 (11)  Leukopenia: grade 4 (16)  Thrombocytopenia: grade 2 (2), grade 3 (2), grade 4 (8) | Herpes zoster: grade 3 (1),  Gum infection: grade 3 (1)  Device infection: grade 3 (1) | Interstitial lung disease: grade 5 (1) | Pancreatitis: grade 4) | Not reported | Grade 4 pancreatitis (1) and Interstitial lung disease (1)  Death from interstitial lung disease (1) |
| Bortezomib (BTZ), dose-limiting toxicities (DLTs), relapsed/refractory (r/r), acute lymphoblastic leukemia (ALL), gastrointestinal (GI), acute myeloid leukemia (AML), antibody-mediated rejection (AMR). | | | | | | | | | |

| **Supplemental Table 6. Toxicities in case reports/case series of pediatric patients treated with BTZ** | | | | | | | | | |
| --- | --- | --- | --- | --- | --- | --- | --- | --- | --- |
| **Study** | **Indication** | **# of evaluable patients** | **Neurological**  **(# of patients)** | **Hematological/Bone Marrow**  **(# of patients)** | **Infections**  **(# of patients)** | **Respiratory**  **(# of patients)** | **Gastrointestinal**  **(# of patients)** | **Cardiac**  **(# of patients)** | **DLTs**  **(# of patients)** |
| Morrow (2012) | AMR cardiac transplant | 4 | None | Transient thrombocytopenia: grade unspecified (1) | None | None | None | None | None |
| Ryckewaert (2013) | AMR renal transplant | 1 | None | Mild transient lymphopenia (1), thrombocytopenia (1), and anemia (1) treated with EPO | None | None | None | None | None |
| Westphal (2013) | AMR renal transplant | 1 | None | None | None | None | None | None | None |
| Twombley (2013) | AMR renal transplant | 2 | None | None | None | None | None | None | None |
| Banugaria (2013) | High sustained antibody titers from ERT in Pompe disease | 3 | None | None | None | None | None | None | None |
| Plant (2013) | Plasmacytoma-like post-transplant lymphoproliferative disorder | 2 | None | None | None | None | None | None | None |
| Zinn (2014) | AMR cardiac transplant | 3 | None | Anemia: grade 3 (2)  Neutropenia: grade 4 (1)  Lymphopenia: grade 4 (2) | Cellulitis (1), Bacteremia (1) | None | Diarrhea: grade 2 (2) | None | Severe leukopenia, BTZ held for one week then reduced to 1 mg/mg^2^ dose (1) |
| Claes (2014) | AMR renal transplant | 1 | None | None | None | None | None | None | None |
| Nguyen (2014) | AMR renal transplant | 4 | None | None |  | None | None | None | None |
| May (2014) | HLA desensitization prior to cardiac transplant | 1 | Neuropathic pain in legs (1) | None | None | None | None | None | Dose reduced due to neuropathic pain, resolved and usual dose was resumed (1) |
| Khandelwal (2014) | Refractory autoimmunity^1^ | 7 | None | Thrombocytopenia: grade 2 (1), grade 3 (1) | Grade unspecified cellulitis (1) | Not reported | Nausea: grade unspecified (1) and grade unspecified *C. difficile* colitis (1) | None | Dose reduced to 1 mg/m^2^ because of thrombocytopenia (1) |
| Waespe (2014) | AIC after HSCT for ALL | 1 | None | None | None | None | None | None | None |
| Mehta (2014) | AIHA after HSCT for SCID | 1 | None | None | None | None | None | None | None |
| Van Balen (2014) | Refractory TTP | 1 | None | None | None | None | None | None | None |
| Preston (2014) | Desensitization prior to lung transplant | 1 | None | None | None | None | None | None | None |
| Hirabayashi (2014) | recurrent ALL | 1 | None | Neutropenia: no grade (1) | None | None | None | None | None |
| Roberti (2015) | AMR renal transplant | 2 | None | None | Acute herpetic angina, CMV, oral and vaginal ulcers due to atypical mycobacteria. All in a single patient (1) | None | None | None | None |
| Kim (2015) | Mucopolysaccharidosis type II | 1 | None | None | None | None | None | None | None |
| Hayes Jr. (2016) | AMR lung transplant | 1 | None | None | None | None | None | None | None |
| Pearl (2016) | AMR renal transplant | 7 | None | None | Bacteremia (1) and mild sinus infection (1) Both recovered with antibiotics | None | Diarrhea: grade unspecified (2) | None | None |
| Pirojsakul (2016) | HLA desensitization prior to renal transplantation | 4 | None | None | None | None | None | None | None |
| Carden (2017) | Metastatic renal medullary carcinoma | 2 | Peripheral neuropathy: grade 3 (1) | Myelosuppression less than grade 3 (1) | None | None | Nausea/vomiting: grade unspecified (1) | None | BTZ maintenance discontinued due to grade 3 peripheral sensory neuropathy of the hands (1) |
| Kulkarni (2017) | Solitary plasmacytoma of bone | 1 | None | None | None | None | None | None | None |
| Kruizinga (2018) | AIC after HSCT | 7 | Not reported | Not reported | Not reported | Not reported | Not reported | None | None |
| Torrealba (2018) | Proliferative glomerulonephritis with monoclonal immunoglobulin G deposits | 1 | None | None | None | None | None | None | None |
| Epperly (2018) | Plasma cell myeloma | 1 | None | None | None | None | None | None | None |
| Tasaki (2019) | AMR/PCAR renal transplant | 3 | None | None | None | None | None | None | None |
| Cordani (2019) | Refractory Anti-NMDA receptor encephalitis | 1 | None | None | None | None | None | None | None |
| Lo Nigro (2019) | recurrent B-ALL | 2 | Peripheral neuropathy: grade 2 (1), spontaneously resolved | None | None | None | None | None | None |
| Knops (2020) | AIHA after intestinal transplant | 1 | None | Leukopenia: ungraded (1)  Thrombocytopenia: ungraded (1) | None | None | Increased ileal stoma output, resolved after cessation of therapy (1) | None | Only 2 doses of second cycle given because of increased ileal stoma output and bone marrow suppression (1) |
| Knight (2020) | Refractory Evans syndrome | 1 | None | None | None | None | None | None | None |
| Beydoun (2020) | Refractory Evans syndrome | 3 | None | None | None | None | None | None | None |
| Maschan (2020) | Refractory TTP | 2 | None | None | None | None | None | None | None |
| Turnbull (2020) | Refractory Anti-NMDA receptor encephalitis | 1 | None | None | None | None | None | None | None |
| Wang (2020) | recurrent B-ALL | 1 | None | None | None | None | None | None | None |
| Azapagasi (2021) | Refractory TTP | 1 | None | None | None | None | None | None | None |
| Simmons (2021) | Refractory Anti-NMDA receptor encephalitis | 1 | None | Mild transient neutropenia (1) | None | None | None | None | None |
| Govil-Dalela (2021) | Refractory Anti-NMDA receptor encephalitis | 1 | None | None | None | None | None | None | None |
| Ryan (2021) | Renal medullary carcinoma | 1 | None | None | None | None | None | None | None |
| Wang (2021) | Multiple myeloma | 1 | None | None | None | None | None | None | None |
| Conti (2022) | Refractory Evans syndrome | 1 | None | None | None | None | None | None | None |
| Ceolin (2022) | recurrent T-ALL | 1 | Guillain-Barré Syndrome (1) | None | None | None | None | None | None |
| Wali (2023) | Refractory TTP | 1 | None | None | None | None | None | None | None |
| Dalela (2023) | Renal medullary carcinoma | 1 | None | None | Pancytopenia (1) | None | Diarrhea (1) | None | None |
| Wang (2023) | recurrent B-ALL with TP53c.C275T mutation | 1 | None | None | None | None | None | None | None |
| Sasaki (2023) | Membranous nephropathy secondary to ERT in Pompe disease | 1 | None | None | None | None | None | None | None |
| Sanai (2023) | GVHD prophylaxis post HSCT | 1 | None | None | None | None | None | None | None |
| Desai (2024) | High sustained antibody titers from ERT in Pompe disease | 2 | None | None | None | Self-limited RSV infection (1) | None | None | None |
| Bortezomib (BTZ), dose-limiting toxicities (DLTs), antibody-mediated rejection (AMR), erythropoietin (EPO), plasma cell-rich acute rejection (PCAR), granulocyte colony stimulating factor (G-CSF), cytomegalovirus (CMV), human leukocyte antigen (HLA), autoimmune cytopenia (AIC), hematopoietic stem cell transplant (HSCT), Graft-versus-host disease (GVHD), acute lymphoblastic leukemia (ALL), autoimmune hemolytic anemia (AIHA), severe combined immunodeficiency (SCID), thrombotic thrombocytopenic purpura (TTP), enzyme replacement therapy (ERT), N-methyl-D-aspartate (NMDA), Autoimmune cytopenia (AIC). | | | | | | | | | |

| **Supplementary Table 7. Summary of toxicities of all grades in pediatric patients treated with bortezomib as a single agent** | | | |
| --- | --- | --- | --- |
| **Indication** | **Evaluable patients** | **# of adverse events** | **% of patients experiencing complication, all grades** |
| **Peripheral Neuropathy** |  |  |  |
|  | 22 | 2 | **9.09%** |
| **Other Neurological** |  |  |  |
|  | 22 | 1 | **4.55%** |
| **Anemia** |  |  |  |
|  | 15 | 6 | **40.00%** |
| **Neutropenia** |  |  |  |
|  | 15 | 4 | **26.67%** |
| **Febrile Neutropenia** |  |  |  |
|  | 7 | 1 | **14.29%** |
| **Thrombocytopenia** |  |  |  |
|  | 20 | 4 | **20.00%** |
| **Leukopenia** |  |  |  |
|  | 15 | 1 | **6.67%** |
| **Lymphopenia** |  |  |  |
|  | 2 | 0 | **0.00%** |
| **Infection** |  |  |  |
|  | 22 | 2 | **9.09%** |
| **Respiratory toxicities, excluding those attributable to infection** | | | |
|  | 7 | 1 | **14.29%** |
| **GI** |  |  |  |
|  | 22 | 7 | **31.82%** |
| **Cardiac** |  |  |  |
|  | 22 | 2 | **9.09%** |
| Acute lymphoblastic leukemia (ALL); acute myeloid leukemia (AML); not reported (NR); anti-NMDA, Anti-N-methyl-d-aspartate. | | | |
